# Eligibility criteria in phase 3 randomized controlled trials in gastric cancer

**DOI:** 10.1007/s10120-025-01653-3

**Published:** 2025-09-11

**Authors:** Katarzyna Marcisz-Grzanka, Danuta Kłosowska, Marek Harhala, Jan Borysowski

**Affiliations:** 1 Department of Clinical Oncology and Radiotherapy, Maria Sklodowska-Curie National Research Institute of Oncology (MSCNRIO), Wawelska 15, 02-034 Warsaw, Poland; 2 Department of Clinical Immunology, Medical University of Warsaw, Nowogrodzka 59, 02-006 Warsaw, Poland; 3 Institute of Human Biology and Evolution, Faculty of Biology, Adam Mickiewicz University, Uniwersytetu Poznanskiego 6, 61-614 Poznan, Poland

**Keywords:** Gastric cancer, Randomized controlled trial, Clinical trial, Eligibility criteria

## Abstract

**Background:**

The purpose of this study was to examine the eligibility criteria in phase 3 randomized controlled trials (RCTs) in gastric cancer.

**Methods:**

The analysis included 207 RCTs of systemic treatments, started between 2009 and 2024, and registered at the WHO International Clinical Trials Registry Platform (ICTRP).

**Results:**

93 (44.9%) trials had an upper age limit of 85 years of age or lower (coprimary outcome). In multivariable analysis, these limits were less likely in RCTs with the sites located in North America (adjusted odds ratio [aOR], 0.06; 95% confidence interval [CI] 0.01–0.26; *p* < 0.001). Only 3 (1.4%) trials were specifically dedicated to older patients. 138 (66.7%) trials excluded patients with Eastern Cooperative Oncology Group (ECOG) score > 1 (coprimary outcome); these criteria were more likely in more recent trials (aOR, 4.49; 95% CI 2.11–9.94; *p* < 0.001). However, the odds of excluding individuals with ECOG score > 1 were not significantly associated with any type of the investigational treatment including chemotherapy (*p* > 0.05). Moreover, many trials excluded patients with brain metastases (*n* = 91; 44%) and those with comorbidities, most frequently liver disorders (*n* = 170; 82.1%). None of the RCTs excluded patients based on frailty.

**Conclusions:**

The eligibility criteria in phase 3 RCTs in gastric cancer are fairly strict. Recommendations presented in this article will allow the investigators to improve the enrollment of some clinically relevant populations of patients, especially older persons, individuals with inadequate performance status, and those with comorbidities, without substantially compromising the safety of trials participants.

**Supplementary Information:**

The online version contains supplementary material available at 10.1007/s10120-025-01653-3.

## Introduction

Gastric cancer (GC) is a major problem influencing life expectancy due to its aggressive nature and has a poor prognosis dependent on the tumor stage at presentation [1, 2]. According to the most recent GLOBOCAN estimates, it is the fifth most common cancer and the fourth leading cause of cancer-related mortality worldwide. GC most commonly occurs over the age of 60, and is twice as frequent in men as in women [3]. The number of older persons with GC, already very high, is expected to grow in the decades to come as a result of the aging of the societies worldwide [4].

Unfortunately, most patients are diagnosed at advanced (either locally advanced or metastatic) stage of GC development. The proper treatment qualification is based on endoscopy, imaging studies, and, in some cases, laparoscopy. The gold standard in therapy of locally advanced GC is perioperative treatment, which involves qualification after pathomorphological assessment of HER-2, Claudin 18.2, PD-L1 expression, and microsatellite instability [5]. The mainstay of the treatment of metastatic GC is systemic chemotherapy. However, the response rate after chemotherapy in both locally advanced and metastatic GC is still limited [6–8], resulting in the median survival time being less than one year and approx. 12 months, respectively [5, 9]. Moreover, it needs to be underscored that assessing nutritional status has become an integral element of modern multidisciplinary oncology treatment due to the impact of this status on treatment tolerance and overall survival in cancer patients [10].

Owing to the low efficacy of the approved treatments for GC, it is very important to conduct clinical trials of new drugs. For the past several decades, the gold standard of study to assess the efficacy and safety of new anticancer drugs has been the phase 3 randomized controlled trial (RCT) [11]. One of the key aspects of an RCT are the eligibility criteria which serve two main purposes. First, they ensure the safety of patients enrolled in a clinical trial. Second, by eliminating different confounding factors, they ensure adequate external validity (generalizability) of the results of clinical trials [12]. However, overly restrictive eligibility criteria may hinder the access of patients to novel drugs, slow down the enrollment process (which may even result in a trial’s premature termination), and substantially limit the generalizability of clinical trials [13]. In view of these consequences, over the last two decades, both major regulatory agencies and professional oncology societies undertook efforts to broaden the eligibility criteria in cancer clinical trials [14–16]. Broadening of the eligibility criteria may considerably improve the diversity of trials participants; in particular, it can optimize the eligibility of some populations of patients that have traditionally been underrepresented in oncology clinical trials [17].

The objective of this study was to examine the eligibility criteria in phase 3 RCTs in GC. To our knowledge, we are the first to provide a comprehensive analysis of these criteria.

## Methods

### Identification of eligible clinical trials

Clinical trials concerning gastric cancer were searched for in the World Health Organization (WHO) International Clinical Trials Registry Platform (ICTRP) [18]. This web-based resource provides access to the data deposited in over 20 primary clinical trials registries including ClinicalTrials.gov as well as the registries of the European Union, China, and Japan. Overall, the resource provides coverage of clinical trials being performed all over the world.

To be eligible for inclusion, a trial had to: (1) be a randomized phase 3 trial; (2) have a protocol involving systemic administration of the investigational drug or biological (either alone or in a combination with other interventions); (3) enroll patients with gastric adenocarcinoma (trials enrolling individuals with gastroesophageal junction adenocarcinoma were also included).

To select eligible trials we queried the search portal of the WHO ICTRP on March 4, 2024. The search string containing a variety of terms related to GC and gastroesophageal junction adenocarcinoma is provided in Additional File. Using ‘Advanced Search’ function of the WHO ICTRP, we narrowed down the search to phase 3 trials registered on or after January 1, 2009. Next, after the removal of duplicates, we manually reviewed all potentially eligible records and excluded studies with the following characteristics: (1) observational studies; (2) nonrandomized clinical trials; (3) trials of non-pharmacological interventions; (4) trials of pharmacological interventions applied locally; (5) trials concerning other diseases and cancer types including gastrointestinal neuroendocrine tumor (NET) and gastrointestinal stromal tumor (GIST); (6) basket trials; (7) trials started before 2009; (8) trials concerning the treatment of complications of gastric cancer; (9) cancer prevention trials; (10) trials with status ‘withdrawn’ or ‘suspended’.

### Data extraction and analysis

From record of each included trial, we imported in XML format a range of data, including the sponsor, the trial’s start and registration date, the sample size, the investigational intervention, the investigational site(s), and the age limits. The data on other eligibility criteria were extracted manually. All data were inserted in an Excel spreadsheet and were analyzed in Excel. The data were extracted and analyzed by one investigator, and another investigator checked the correctness of the data. All discrepancies were resolved through consensus.

Coprimary outcomes of this study were the proportion of trials with an upper age limit of 85 years or lower and the proportion of RCTs excluding patients with Eastern Cooperative Oncology Group (ECOG) score > 1. Secondary outcomes included the proportions of RCTs with other eligibility criteria, especially those concerning brain metastases, comorbidities, and frailty.

### Statistical analysis

All included trials were characterized descriptively. The variables affecting the odds of the upper age limits and of the eligibility criteria involving the performance status of the patient were identified by multivariable logistic regression with Firth biased reduction method. Threshold for the calculation of the confusion matrix was optimized and confusion matrices are reported in Additional File (Additional Table 1 and Additional Table 2). The results of logistic regression analyses were reported as adjusted odds ratios (aORs) with 95% confidence intervals (CIs).

The trends in the use of the upper age limits and the eligibility criteria involving the performance status over time were examined by Cochran–Armitage test for trend. Temporal trends in the use of the eligibility criteria related to selected comorbidities and brain metastases were assessed by Pearson’s Chi-squared test with Yates’ continuity correction. To that end, we compared the proportion of RCTs with a given criterion between the trials started from 2009 to 2016 and those initiated between 2017 and 2024. False discovery rates in Chi-squared tests were controlled for using Benjamini and Hochberg method.

All statistical analyses were performed in R programming language (logistf and DescTools packages for logistic regression and Cochran–Armitage test for trend, respectively) [19]. *p* values less than 0.05 were deemed statistically significant.

## Results

### Characteristics of included trials

**Fig. 1:**
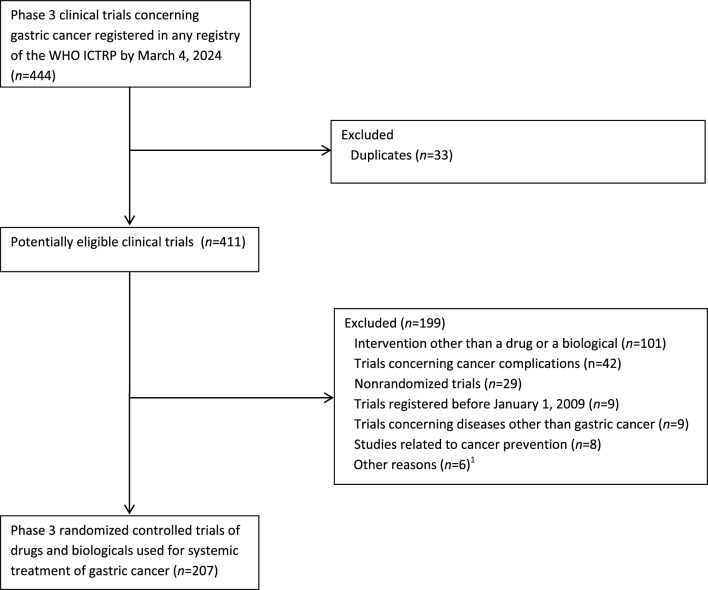
Selection of phase 3 randomized controlled trials of pharmacological interventions used for systemic treatment of gastric cancer. ^1^Including trials with status ‘withdrawn’ or ‘suspended’ (*n* = 2), trials of pharmacological interventions administered topically (*n* = 1), trials concerning gastrointestinal neuroendocrine tumors (NET; *n* = 1), trials related to gastrointestinal stromal tumors (GIST; *n* = 1), and basket trials (*n* = 1). *ICTRP* International Clinical Trials Registry Platform

Selection of eligible RCTs is shown in Fig. 1. The initial search in the WHO ICTRP yielded 444 trials. The most common reason for a trial’s exclusion was the use of an intervention other than a drug or a biological (*n* = 101). Eventually, 207 phase 3 RCTs met all the eligibility criteria and were selected for analysis. Included trials are listed in Additional File and their characteristics are shown in Table 1.

**Table 1:** Characteristics of phase 3 randomized controlled trials in gastric cancer

	*n*	%
Line of treatment		
1	84	40.6
≥2	43	20.8
Perioperative treatment	74	35.7
Maintenance treatment^a^	6	2.9
Investigational intervention		
Chemotherapy	90	43.5
Chemotherapy + immunotherapy	34	16.4
Chemotherapy + targeted drug	27	13.0
Chemotherapy + HIPEC	11	5.3
Targeted drug	11	5.3
Immunotherapy	9	4.3
Other	25	12.7
Investigational site		
Asia	139	67.1
Europe	21	10.1
North America	4	1.9
Other	2	1
Intercontinental	41	19.8
Sample size		
≤99	54	26.1
100–499	64	30.9
≥500	88	42.5
Missing data	1	0.5
Primary sponsor		
Non-commercial	115	44.4
Pharmaceutical industry	92	55.6

*HIPEC* hyperthermic intraperitoneal chemotherapy

^a^Maintenance treatment following the completion of the first-line treatment. Percentages may not add to 100% due to rounding error

The investigational regimens assessed in included trials commonly involved chemotherapy (*n* = 90; 43.5%), and chemotherapy in a combination with immunotherapy (*n* = 34; 16.4%) or with a targeted drug (*n* = 27; 13.0%). Many RCTs (*n* = 84; 40.6%) evaluated the use of the investigational intervention as the first-line treatment. Most trials were conducted in the investigational sites located solely in Asia (*n* = 139; 67.1%). Our sample also involved many RCTs (*n* = 41; 19.8%) with the sites located in more than one continent. 116 (54.7%) trials had non-commercial sponsors. The largest proportion of trials (*n* = 88; 42.5%) comprised RCTs with the sample sizes ≥ 500.

### Eligibility criteria

#### Upper age limits

Overall, as many as 99 trials (47.8%) had an upper age limit (Table 2). The most frequent limit, used in 56 (27.1%) RCTs, was 75 years of age. 93 (44.9%) trials listed an upper age limit of 85 years or lower (coprimary outcome). Multivariable logistic regression (Table 3) showed that the odds of an upper age limit of 85 years or lower were higher in RCTs enrolling patients with early/locally advanced resectable GC relative to metastatic GC (aOR, 2.38; 95% CI 1.11–5.21; *p* = 0.02) and lower in RCTs with the sites located in North America (aOR, 0.06; 95% CI 0.01–0.26; *p* < 0.001). However, none of the other variables including the investigational intervention type and the sponsor type significantly affected the odds of excluding older adults (*p* > 0.05 for each variable; Table 3).

**Table 2:** Eligibility criteria in phase 3 randomized controlled trials in gastric cancer

	*n*	%
Lower age limits		
18–29	184	88.9
30–59	1	0.5
60–69	0	0
≥70	3	1.4
No limit	19	9.2
Upper age limits		
≤ 65	2	1.0
66–70	22	10.6
71–75	58	28.0
76–80	11	5.3
81–85	0	0
>85	6	2.9
No limit	108	52.2
Performance status		
Exclusion of ECOG ≥ 2	138	66.7
Exclusion of ECOG ≥ 3	61	29.5
No limit	8	3.9
Comorbidities		
Liver diseases	170	82.1
Kidney diseases	168	81.2
Bone marrow function	157	75.8
Malignancies	147	71.0
Cardiac diseases	141	68.1
Psychiatric disorders	75	36.2
Pulmonary diseases	74	35.7
Hypertension	70	33.8
HIV infection	57	27.5
Autoimmune diseases	44	21.3
Stroke	36	17.4
Thromboembolic diseases	23	11.1
IBD	21	10.1
Brain metastases		
Active^a^	53	25.6
Any	38	18.4
Frailty	0	0

Trials may have had multiple eligibility criteria

*ECOG* Eastern Cooperative Oncology Group, *HIV* human immunodeficiency virus, *IBD* inflammatory bowel disease

^a^Including metastases classified as ‘active’, ‘uncontrolled’, ‘unstable’, ‘untreated’, ‘symptomatic’, and ‘progressive’

**Table 3:** Trials characteristics affecting the odds of the upper age limits and the eligibility criteria related to the performance status of the patient

	Performance status^a^		Upper age limits^b^	
	Adjusted odds ratio (95% CI)	*p*	Adjusted odds ratio (95% CI)	*p*
Cancer stage				
Metastatic	Referent	–	Referent	–
Early/LAR	1.74 (0.79–3.93)	0.16	2.38 (1.11–5.21)	0.02
Start date				
2009–2016	Referent	–	Referent	–
2017–2024	4.49 (2.11–9.94)	<0.001	1.60 (0.77–3.39)	0.20
Site in North America^c^				
No	Referent	–	Referent	–
Yes	1.85 (0.54–6.69)	0.32	0.06 (0.01–0.26)	<0.001
Site in Asia^c^				
No	Referent	–	Referent	–
Yes	2.18 (0.61–7.86)	0.22	1.33 (0.25–6.74)	0.72
Site in Europe^c^				
No	Referent	–	Referent	–
Yes	3.43 (0.97–12.84)	0.06	0.37 (0.07–1.74)	
Chemotherapy				
No	Referent	–	Referent	–
Yes	0.32 (0.08–1.07)	0.06	0.97 (0.33–2.88)	0.95
Targeted drug				
No	Referent	–	Referent	–
Yes	2.44 (0.95–6.83)	0.06	1.37 (0.56–3.45)	0.49
Immunotherapy				
No	Referent	–	Referent	–
Yes	1.23 (0.42–3.78)	0.71	2.10 (0.72–6.62)	0.17
Sample size				
≤249	Referent	–	Referent	–
250–499	0.90 (0.37–2.15)	0.81	1.24 (0.53–2.95)	0.61
≥500	1.17 (0.48–2.83)	0.72	1.15 (0.48–2.78)	0.75
Primary sponsor				
Commercial	Referent	–	Referent	–
Non-commercial	0.78 (0.30–2.07)	0.61	2.19 (0.85–5.87)	0.10

^a^Eligibility criteria excluding patients with ECOG > 1

^b^Upper age limit of 85 years or lower

^c^Denotes clinical trials with at least one investigational site located in a given continent

*CI* confidence interval, *LAR* locally advanced resectable

To verify the robustness of the findings from the primary analysis, we performed three sensitivity analyses. In the first analysis, for each trial, we replaced the trial’s start date with the registration date. In the second analysis, we employed a different categorization of the sample size; rather than divide the sizes into three categories as was the case in the primary analysis (≤99; 100–499; and ≥500), we used a dichotomous division for this variable (<500 and ≥500). In the third sensitivity analysis, we assessed how the variables used in the primary analysis affect the odds of the presence of an upper age limit of 75 years or lower. All three analyses had very similar results; each of them confirmed that the odds of an upper age limit were significantly lower in the trials with at least one North America-based site (*p* < 0.001 for each analysis; Additional Tables 3–5, Additional File). However, they failed to confirm a statistically significant association between the GC stage and the presence of an upper age limit (*p* > 0.05 for each analysis; Additional Tables 3–5, Additional File). Thus, overall, the variable with the strongest association with the upper age limits is the investigational site location, especially the presence of the North America-based sites (these mostly included the sites located in the USA; detailed data not shown).

We also showed a statistically significant trend to increasing the proportion of trials with an upper age limit of 85 years or lower over time in analyses involving both the trial’s start date (average change/year 0.02; 95% CI 0.008–0.03; *p* = 0.003) and the registration date (average change/year 0.02; 95% CI 0.009–0.04; *p* = 0.001).

Moreover, we found that our sample included only 3 (1.4%) trials specifically dedicated to older patients. The lower age limit in one of these RCTs was 70 years, and two others enrolled patients aged 80 years or older. In addition, one trial (0.5%) was dedicated to unfit patients aged 18 or older. None of these four trials had an upper age limit.

#### Performance status of the patient

The eligibility criteria involving the performance status of the patient were used in 199 (96.1%) RCTs. Most trials (*n* = 138; 66.7%) allowed only for the enrollment of patients with ECOG score of 0/1. The odds of excluding individuals with ECOG score > 1 were higher in RCTs started between 2017 and 2024 compared with those begun between 2009 and 2016 (aOR, 4.49; 95% CI 2.11–9.94; *p* < 0.001). We also noted that none of the investigational intervention types including chemotherapy or the sponsor type had any statistically significant association with the odds of excluding individuals with ECOG score > 1 (*p* > 0.05 for each variable; Table 3).

These results were verified by two sensitivity analyses involving some modifications of the variables used in the primary logistic regression model. Both analyses confirmed that the odds of excluding patients with ECOG score > 1 were significantly higher in more recent trials compared with earlier RCTs (*p* < 0.001 for each analysis; Additional Tables 6 and 7, Additional File).

Likewise, the year-by-year analyses showed a statistically significant trend to increasing the proportion of trials excluding patients with ECOG score > 1 over time, both for the trial’s start date (average change/year 0.04; 95% CI 0.02–0.05; *p* < 0.001) and the registration date (average change/year 0.04; 95% CI 0.03–0.05; *p* < 0.001).

### Eligibility criteria involving comorbidities

Full list of the eligibility criteria involving comorbidities of different organs and systems is provided in Additional File. The most common criteria concerned liver disorders (*n* = 170; 82.1%). The most frequent criterion referring to liver function involved aminotransferase concentration relative to upper limit of normal (ULN; *n* = 105; 50.7%; median cut-off, 2.5 ULN; range, 1.5–5 ULN). The proportion of trials excluding patients with liver disorders was comparable between the recent and earlier RCTs (52.9 vs. 47.1%; *p* = 0.28).

Many trials (*n* = 168; 81.2%) excluded patients with renal impairment, frequently based on creatinine concentration relative to ULN (*n* = 68; 32.9%; median cut-off, 1.5 ULN; range, 1–2 ULN). We found no statistically significant difference in the proportion of trials excluding patients with renal impairment between the recent and earlier RCTs (53.6 vs. 46.4%; *p* = 0.40). The proportion of trials with very strict eligibility criteria concerning renal function (the cut-off for creatinine clearance 60 or creatinine level ≤ 1.0 ULN) was clearly lower among recent trials (39%) compared with earlier RCTs (61%). However, this difference fell short of statistical significance (*p* = 0.08). A similar result was found in an analysis performed for the trials with slightly relaxed eligibility criteria (the cut-off for creatinine clearance 45–60 or the cut-off for creatinine level 1.0–1.25 ULN)—41 vs. 59% (*p* = 0.056).

Other commonly used eligibility criteria concerned bone marrow function (*n* = 157; 75.8%). The proportion of trials excluding patients with bone marrow dysfunction was comparable between the recent and earlier RCTs (53.5 vs. 46.5%; *p* = 0.24).

The results of the analyses of trends in the use of the eligibility criteria concerning some comorbidities over time were verified by sensitivity analyses performed by the trial’s registration date. These had very similar results (Additional Table 8, Additional File), thus confirming the results of the primary analyses performed by the trial’s start date.

### Eligibility criteria concerning brain metastases

Overall, the eligibility criteria related to brain metastases were listed in 91 (44%) RCTs. 38 trials (18.4%) excluded patients with any form of brain metastases (strict exclusion), while 53 (25.6%) RCTs excluded individuals with active, symptomatic, or untreated brain metastases (conditional exclusion). In analysis by the trial’s start date, the overall proportion of trials excluding patients with brain metastases was comparable between the recent and earlier RCTs (49.5 vs. 50.5%; *p* = 0.65). However, the proportion of RCTs with strict exclusion was significantly higher among earlier trials compared with recent trials (73.7 vs. 26.3%; *p* < 0.001). By contrast, conditional exclusion of patients with brain metastases was significantly more common in recent RCTs compared with earlier trials (66 vs. 34%; *p* = 0.01). Very similar results were obtained in a sensitivity analysis performed for the trial’s registration date (Table S9, Supporting Information).

### Eligibility criteria related to frailty

None of the trials (0%) had any eligibility criteria related to frailty.

## Discussion

Our study showed that most phase 3 RCTs in GC exclude clinically relevant populations of patients. First, 44.9% of the trials had an upper age limit of 85 years or lower. This is an important problem, because a considerable proportion of patients with GC are older adults [20]. In view of some important differences regarding the biology of GC [21, 22], comorbidities [23], and other factors, the results obtained in younger patients may not be generalizable to older individuals. Besides, it needs to be underscored that older patients with cancer comprise a very heterogeneous population with respect to their age, comorbidities, frailty status, cognitive abilities, and social support; therefore, the chronological age of an older person does not necessarily correlate with his/her functional status and should not be used as an exclusion criterion in oncology clinical trials [24]. Comprehensive geriatric assessment (CGA) is recommended to assess the functional state of an older patient with GC [25]. A cheaper and less time-consuming alternative to CGA are some validated tools that can also be employed to identify frail individuals [26]. Regretfully, none of the trials from our sample had any frailty-related eligibility criteria.

We also found that only 3 RCTs were dedicated to older adults. In our view, more such trials are needed, especially owing to widespread excluding of older patients from ‘standard’ phase 3 trials in GC. Using proper eligibility criteria, these RCTs might be designed to enroll the populations of patients who are most underrepresented in clinical trials, especially the oldest-old individuals (i.e., those older than 80 or 85 years) and frail patients (the prevalence of frailty among patients with GC is estimated to be 29%) [27]. They might also utilize the endpoints that are more relevant to older patients [28, 29].

One of the most common eligibility criteria in the trials from our sample involved the performance status of the patient, with most RCTs excluding individuals with ECOG score > 1. The use of the scales such as the ECOG for the assessment of the performance status of the patient in cancer clinical trials has some significant limitations. First, these scales were developed several decades ago and their reliability in the toxicity risk stratification of newer anticancer treatments may be inadequate [30]. Second, they are based on subjective assessment of the state of the patient and, therefore, are prone to bias [31]. Third, the prognostic value of the performance status among patients with ECOG score of 0–2 can be limited [32]. Moreover, the scales used for the assessment of the performance status are not able to differentiate patients in whom the reduced status is caused by cancer from those in whom it is related to other factors such as severe comorbidities. This is an important problem, because the former group comprise patients who may derive benefits from anticancer treatment [15]. It should be also noted that patients with GC and ECOG score of 2 have different baseline characteristics compared with those with ECOG score of 0/1 [33]; therefore, the results obtained in patients with optimal performance score may not be generalizable to those with ECOG score of 2.

We also observed a clear difference in the pattern of the two key types of the eligibility criteria between trials with the investigational sites located solely in Asian countries and those with no Asian sites. Age-based exclusions were much more common in Asian trials (86/139 trials; 61.9%) compared with non-Asian trials (9/68; 13.2%). By contrast, excluding patients with ECOG score > 1 was more frequent in non-Asian trials (53/68; 77.9%) compared with Asian trials (85/139; 61.2%). Thus, trials performed in Asian countries commonly exclude patients based on the upper age limits, while non-Asian trials tend to exclude based on inadequate performance score of the patient.

Moreover, we found that the vast majority of the trials excluded patients with different comorbidities. The most common eligibility criteria related to comorbidities involved liver disorders, renal diseases, and bone marrow function. These criteria also substantially limit the external validity of the trials, because most ‘real-world’ patients with GC have comorbidities [34]. For instance, one study showed that approx. 80% of the patients with GC had moderate- or high-risk Charlson Comorbidity Index (CCI) score. Furthermore, the CCI score increased with the patients’ age [23]. Thus the eligibility criteria related to comorbidities may particularly increase the risk of excluding older patients with GC.

For instance, many trials from our sample had very strict exclusion criteria related to renal function, especially creatinine clearance 50 or 60 mL/min and creatinine concentration 1 or 1.25 ULN. The rationale for this is likely a fact that many chemotherapeutics used in GC are potentially nephrotoxic and the risk of nephrotoxicity may be higher in patients with pre-existing renal impairment. However, rather than exclude patients with mild or moderate kidney disease, a chemotherapeutic’s dose reduction may be considered to minimize the likelihood of nephrotoxicity. In fact, the dose adjustment recommendations for many chemotherapeutics were developed and may be employed in patients with mild and moderate kidney decline [35]. A need for wider enrollment of patients with renal impairment to oncology clinical trials has also been suggested by other authors [36, 37].

Many trials from our sample excluded patients with brain metastases. This is likely caused by a dismal prognosis of such patients [38]. We believe that a lack of effective treatments implies a need for wider enrollment of patients with brain metastases to clinical trials in GC. In our opinion, especially patients with treated/stable metastases should be enrolled. Therefore, an encouraging finding was a growing proportion of trials allowing for the enrollment of patients with treated/stable brain metastases in our sample. By contrast, the number of RCTs excluding individuals with any form of brain metastases was low and clearly decreasing over time.

Based on the results of our analysis, we propose a number of recommendations to broaden the eligibility criteria in GC. First, the upper age limits should not be used; the actual functional status of an older patient should be assessed be performing CGA or by using a simpler validated tool. Second, more trials should be open to patients with ECOG score of 2, provided that the investigational drug has acceptable toxicity profile and inadequate performance status is caused by cancer itself and not by other factors such as severe comorbidities. Moreover, the relaxation of some of the eligibility criteria related to comorbidities should be considered, especially strict criteria concerning renal function.

The strength of this study is that, to our knowledge, we are the first to perform a comprehensive analysis of the eligibility criteria in phase 3 RCTs in GC. The main limitation is that we had no access to full trials protocols and therefore had to rely on the data from the registries. Another limitation is that we focused on phase 3 trials and did not examine trials of other phases, especially phase 2 trials, which may also provide important data on the efficacy and safety of the investigational drugs.

## Conclusions

In conclusion, many phase 3 trials in GC have strict eligibility criteria hindering the enrollment of some clinically relevant populations of patients. Careful relaxation of some of these criteria will improve the external validity of the trials without overly compromising the safety of their participants.

## Supplementary Information

Below is the link to the electronic supplementary material.

Supplementary material 1. Additional Table 1. Multiple metrics describing performance of logistic regression model presented in Table 3, ‘Performance status’ columns, docx. Additional Table 2. Multiple metrics describing performance of logistic regression model used in Table 3, ‘Upper age limit’ columns, docx. List of phase 3 randomized controlled trials in gastric cancer, docx. Additional Table 3. Variables affecting the odds of the presence of an upper age limit of 85 years or lower in phase 3 randomized controlled trials in gastric cancer (sensitivity analysis replacing the trial’s start date with the registration date), docx. Additional Table 4. Variables affecting the odds of the presence of an upper age limit of 85 years or lower in phase 3 randomized controlled trials in gastric cancer (sensitivity analysis for variable ‘sample size’), docx. Additional Table 5. Variables affecting the odds of the presence of an upper age limit of 75 years or lower in phase 3 randomized controlled trials in gastric cancer (sensitivity analysis for the upper age limits of 85 years or lower), docx. Additional Table 6. Variables affecting the odds of excluding patients with ECOG score > 1 in phase 3 randomized controlled trials in gastric cancer (sensitivity analysis for variable ‘sample size’), docx. Additional Table 7. Variables affecting the odds of excluding patients with ECOG score > 1 in phase 3 randomized controlled trials in gastric cancer (sensitivity analysis replacing the trial’s start dates with the registration date), docx. Eligibility criteria concerning comorbidities in phase 3 randomized controlled trials in gastric cancer, docx. Additional Table 8. Trends in the use of the eligibility criteria concerning selected comorbidities over time (sensitivity analysis by the trial’s registration date), docx. Additional Table 9. Trends in the use of the eligibility criteria concerning brain metastases over time (sensitivity analysis by the trial’s registration date), docx
