# Supplementary material for: Eligibility criteria in phase 3 randomized controlled trials in gastric cancer
Source: Gastric Cancer. 2025 Sep 11;28(6):1232–40. doi: 10.1007/s10120-025-01653-3 (PMC12630207; doi:10.1007/s10120-025-01653-3)
Supplement: Supplementary file 1 — Supplementary material 1. Additional Table 1. Multiple metrics describing performance of logistic regression model presented in Table 3, ‘Performance status’ columns, docx. Additional Table 2. Multiple metrics describing performance of logistic regression model used in Table 3, ‘Upper age limit’ columns, docx. List of phase 3 randomized controlled trials in gastric cancer, docx. Additional Table 3. Variables affecting the odds of the presence of an upper age limit of 85 years or lower in phase 3 randomized controlled trials in gastric cancer (sensitivity analysis replacing the trial’s start date with the registration date), docx. Additional Table 4. Variables affecting the odds of the presence of an upper age limit of 85 years or lower in phase 3 randomized controlled trials in gastric cancer (sensitivity analysis for variable ‘sample size’), docx. Additional Table 5. Variables affecting the odds of the presence of an upper age limit of 75 years or lower in phase 3 randomized controlled trials in gastric cancer (sensitivity analysis for the upper age limits of 85 years or lower), docx. Additional Table 6. Variables affecting the odds of excluding patients with ECOG score > 1 in phase 3 randomized controlled trials in gastric cancer (sensitivity analysis for variable ‘sample size’), docx. Additional Table 7. Variables affecting the odds of excluding patients with ECOG score > 1 in phase 3 randomized controlled trials in gastric cancer (sensitivity analysis replacing the trial’s start dates with the registration date), docx. Eligibility criteria concerning comorbidities in phase 3 randomized controlled trials in gastric cancer, docx. Additional Table 8. Trends in the use of the eligibility criteria concerning selected comorbidities over time (sensitivity analysis by the trial’s registration date), docx. Additional Table 9. Trends in the use of the eligibility criteria concerning brain metastases over time (sensitivity analysis by the trial’s registration date), docx [file 10120_2025_1653_MOESM1_ESM.docx]

**Additional File**

**Table of contents**

1. Search string, page 3
2. Additional Table 1, Multiple metrics describing performance of logistic regression model presented in Table 3, ‘Performance status’ columns, page 4
3. Additional Table 2, Multiple metrics describing performance of logistic regression model used in Table 3, ‘Upper age limit’ columns, page 5
4. List of phase 3 randomized controlled trials in gastric cancer, page 6
5. Additional Table 3. Variables affecting the odds of the presence of an upper age limit of 85 years or lower in phase 3 randomized controlled trials in gastric cancer (sensitivity analysis replacing the trial’s start date with the registration date), page 45
6. Additional Table 4. Variables affecting the odds of the presence of an upper age limit of 85 years or lower in phase 3 randomized controlled trials in gastric cancer (sensitivity analysis for variable ‘sample size’), page 47
7. Additional Table 5. Variables affecting the odds of the presence of an upper age limit of 75 years or lower in phase 3 randomized controlled trials in gastric cancer (sensitivity analysis for the upper age limits of 85 years or lower), page 49
8. Additional Table 6. Variables affecting the odds of excluding patients with ECOG score > 1 in phase 3 randomized controlled trials in gastric cancer (sensitivity analysis for variable ‘sample size’), page 51
9. Additional Table S. Variables affecting the odds of excluding patients with ECOG score > 1 in phase 3 randomized controlled trials in gastric cancer (sensitivity analysis replacing the trial’s start dates with the registration date), page 53
10. Eligibility criteria concerning comorbidities in phase 3 randomized controlled trials in gastric cancer, page 55
11. Additional Table 8. Trends in the use of the eligibility criteria concerning selected comorbidities over time (sensitivity analysis by the trial’s registration date), page 61
12. Additional Table 9. Trends in the use of the eligibility criteria concerning brain metastases over time (sensitivity analysis by the trial’s registration date), page 62

**Search string**

Gastric cancer OR Gastric carcinoma OR Gastric adenocarcinoma OR Gastric neoplasm OR Gastric neoplasms OR Gastric tumor OR Gastric tumors OR Stomach cancer OR Stomach neoplasm OR Stomach neoplasms OR Stomach tumor OR Stomach tumors OR Stomach carcinoma OR Stomach adenocarcinoma OR Cancer of stomach OR Neoplasm of stomach OR Gastroesophageal junction cancer OR Gastroesophageal junction carcinoma OR Gastroesophageal junction adenocarcinoma

**Additional Table 1**. Multiple metrics describing performance of logistic regression model presented in Table 3, ‘Performance status’ columns. Metrics are shown as delivered by confusionMatrix() function from caret 7.0 package in R programming language. Threshold for metrics evaluation was optimized for lowest p-value [Acc > NIR] reported in the last row. Abbreviations: CI, confidence interval; NIR, no information rate.

| Accuracy | 0.772 |
| --- | --- |
| Accuracy (95% CI lower limit) | 0.708 |
| Accuracy (95% CI upper limit) | 0.827 |
| No Information Rate | 0.670 |
| P-Value [Acc > NIR] | 0.000893 |
| Kappa | 0.431 |
| Mcnemar's Test P-Value | 0.000464 |
| Sensitivity | 0.471 |
| Specificity | 0.920 |
| Positive Predictive Value | 0.744 |
| Negative Predictive Value | 0.779 |
| Prevalence | 0.330 |
| Detection Rate | 0.155 |
| Detection Prevalence | 0.209 |
| Balanced Accuracy | 0.695 |
| 'Positive' Class | 0 |
| Threshold | 0.395 |

**Additional Table 2**. Multiple metrics describing performance of logistic regression model used in Table 3, ‘Upper age limit’ columns. Metrics are shown as delivered by confusionMatrix() function from caret 7.0 package in R programming language. Threshold for metrics evaluation was optimized for lowest p-value [Acc > NIR] reported in the last row. Abbreviations: CI, confidence interval; NIR, no information rate.

| Accuracy | 0.762 |
| --- | --- |
| Accuracy (95% CI lower limit) | 0.698 |
| Accuracy (95% CI upper limit) | 0.819 |
| No Information Rate | 0.539 |
| P-Value [Acc > NIR] | 2.85E-11 |
| Kappa | 0.528 |
| Mcnemar's Test P-Value | 0.0101 |
| Sensitivity | 0.694 |
| Specificity | 0.842 |
| Positive Predictive Value | 0.837 |
| Negative Predictive Value | 0.702 |
| Prevalence | 0.539 |
| Detection Rate | 0.374 |
| Detection Prevalence | 0.447 |
| Balanced Accuracy | 0.768 |
| 'Positive' Class | 0 |
| Threshold | 0.485 |

**Phase 3 randomized controlled trials in gastric cancer**

| **Trial ID** | **Scientific_title** |
| --- | --- |
| NCT06238843 | A Multicenter, Randomized, Open-label, Phase 3 Study of IBI343 Monotherapy Versus Treatment of Investigator's Choice in Subjects With Previously Treated, Claudin (CLDN) 18.2-positive, HER2-negative, Locally Advanced, Unresectable or Metastatic Gastric or Gastroesophageal Junction Adenocarcinoma |
| NCT06221748 | A Randomized,Multicenter, Open-Label,Phase II/III Study to Evaluate the Safety and Efficacy of Disitamab Vedotin Combined With Cadonilimab in Subjects With HER2-expressing Locally Advanced or Metastatic Gastric Cancer and Gastroesophageal Junction Adenocarcinoma Who Have Progressed on r First-line Therapy |
| NCT06203600 | Randomized Phase II/III Trial of 2nd Line Nivolumab + Paclitaxel + Ramucirumab Versus Paclitaxel + Ramucirumab in Patients With PD-L1 CPS &gt;/= 1 Advanced Gastric and Esophageal Adenocarcinoma (PARAMMUNE) |
| NCT06206733 | A Phase III Study Evaluating the Efficacy and Safety of ASKB589 Combined With CAPOX and PD-1 Inhibitor in Cldn18.2 Postive Patients With Advanced,Recurrent or Metastatic Gastric or Gastroesophageal Junction Adenocarcinoma |
| NCT06177041 | A Phase 3, Multi-Center, Double-Blind, Randomized, Efficacy and Safety Study of M108 Monoclonal Antibody Plus CAPOX Versus Placebo Plus CAPOX as First-line Treatment for Claudin (CLDN) 18.2-Positive, HER2-Negative, PD-L1 CPS<5, Locally Advanced Unresectable or Metastatic Gastric or Gastroesophageal Junction (GEJ) Adenocarcinoma. |
| NCT06123494 | A Phase 3, Multicenter, Randomized, Open-label Study of SHR-A1811 (HER2-ADC) Compared With the Chemotherapy Treatment Chosen by the Investigators for Subjects With HER2-positive Metastatic and/or Unresectable Gastric Cancer or Gastroesophageal Junction Adenocarcinoma Who Have Progressed on or After First-line Anti-HER2 Therapy-containing Regimen |
| ChiCTR2300076761 | Efficacy and Safety of Cadonilimab and COX-2 Inhibitor Combined with Oxaliplatin and Capecitabine (XELOX) in Perioperative Therapy for Locally Advanced Resectable Gastric Adenocarcinoma: A Prospective, Single-Center, Randomized Clinical Trial |
| ChiCTR2300076276 | The Safety and Efficacy of Low-dose Apatinib Combined with PD-1 Antibody and Reduced-dose FLOT Regimen versus PD-1 Antibody Combined with Reduced-dose FLOT as Neoadjuvant Treatment for Locally Advanced Gastric or Gastroesophageal Junction Adenocarcinoma: A Prospective, Multi-center, Phase III, Randomized Controlled Study |
| NCT06093425 | A Phase 3, Randomized, Double-blind, Placebo-controlled Study Evaluating Combination of TST001, Nivolumab and Chemotherapy as First-Line Treatment in Subjects With Claudin18.2 Positive Locally Advanced or Metastatic Gastric or Gastroesophageal Junction (Gastric/GEJ) Adenocarcinoma |
| ChiCTR2300075651 | A prospective, randomized, controlled clinical study of arterial perfusion chemotherapy sequential PD-1 monoclonal antibody conversion in the treatment of advanced (oligometastasis) gastric cancer |
| NCT06028737 | Total Neoadjuvant Chemotherapy With 5-fluoruracil, Leucovorin, Oxaliplatin and Docetaxel in Locally Advanced Gastric and Gastroesophageal Junction Cancer (OCTASUR): Randomized, Single Center, Open Label Trial, Phase 2/3 |
| NCT05978050 | Nimotuzumab Combined With Paclitaxel as Second-line Treatment for Recurrent Metastatic Gastric or Esophagogastric Junction Adenocarcinoma With EGFR Over-expression: A Randomized, Double-blind, Placebo-controlled Phase III Clinical Trial |
| NCT05980481 | A Study of RC48-ADC Combine With Toripalimab and Chemotherapy or RC48-ADC Combine With Toripalimab and Herceptin as First-line Treatment in Local Advanced or Metastatic Gastric Cancer With the HER2 Expression |
| ChiCTR2300072891 | Helicobacter pylori Eradication Combined with Adjuvant Chemotherapy versus Adjuvant Chemotherapy in Locally Advanced Helicobacter pylori Positive Gastric Cancer Patients: An Open-label, Randomized, Controlled Phase III Trial |
| NCT05918094 | Comparing Modified XELOX Plus Sintilimab With Standard XELOX Plus Sintilimab in First-line Treatment for HER2-negative Gastric/Gastroesophageal Junction Adenocarcinoma |
| ChiCTR2300071906 | BC001 combined with paclitaxel versus placebo combined with paclitaxel in the treatment of advanced gastric or gastroesophageal junction adenocarcinoma that has failed first-line standard therapy: a randomized, double-blind, parallel-controlled Phase III study |
| NCT05914610 | Envollizumab Combined With Fruquintinib and SOX Versus SOX for Conversion Therapy in Her-2 Negative Unresectable Locally Advanced Gastric Cancer: a National Multicenter Randomized Controlled Study |
| JPRN-jRCT2051220179 | A Randomized, Open-Label, Multicenter Phase 3 Trial of Domvanalimab, Zimberelimab, and Chemotherapy Versus Nivolumab and Chemotherapy in Participants with Previously Untreated Locally Advanced Unresectable or Metastatic Gastric, Gastroesophageal Junction, and Esophageal Adenocarcinoma - STAR-221 |
| NCT05919381 | A Randomized, Double-blind, Parallel Controlled Phase III Study of Gentuximab Injection Combined With Paclitaxel Injection Versus Placebo Combined With Paclitaxel Injection for Advanced Gastric or Gastroesophageal Junction Adenocarcinoma. |
| NCT05677490 | Randomized Phase III Trial of mFOLFIRINOX vs. FOLFOX With Nivolumab for First-Line Treatment of Metastatic HER2- Gastroesophageal Adenocarcinoma |
| NCT05593458 | A Multicenter, Randomized, Controlled Study of S-1 Combined With Oxaliplatin by Arterial Infusion Plus PD-1 Antibody Versus Conventional SOX Chemotherapy Plus PD-1 Antibody for Locally Advanced Gastric Cancer |
| NCT05610332 | Clinical Efficacy of Albumin Paclitaxel Plus Carrelizumab Versus FLOT in Neoadjuvant Treatment of Locally Advanced Gastric Cancer With Different Immunotypes--A Multi-center, Randomised, Open, Phase 3 Clinical Trial |
| NCT05427383 | A Randomized, Multicenter, Phase ?/? Clinical Study to Evaluate the Efficacy of KN026 in Combination With Chemotherapy in Subjects With HER2 Positive Advanced Unresectable or Metastatic Gastric Cancer (Including Gastro-esophageal Junction Adenocarcinoma) Who Have Failed First-line Therapy. |
| NCT05300945 | HIPEC Combined Gastrectomy in Patients With Advanced Gastric Cancer Received Neoadjuvant Chemotherapy |
| NCT05264896 | Perioperative FLOT Versus Adjuvant XELOX for Locally Advanced Gastric Cancer - a Randomized Controlled Study |
| NCT05149807 | A Randomized, Double-Blind, Multi-Center Phase II/III Clinical Study of PD-L1 Antibody/TGF-ßRII (SHR-1701) Plus Tegafur Gimeracil Oteracil Potassium and Oxaliplatin Versus Placebo Plus Tegafur Gimeracil Oteracil Potassium and Oxaliplatin as Perioperative Treatment in Subjects With Resectable Gastric Cancer or Gastroesophageal Junction Cancer |
| NCT05180734 | An International, Multicenter, Randomized, Double-blind, Phase III Clinical Study to Evaluate the Efficacy and Safety of Toripalimab Injection Combined With Postoperative Adjuvant Chemotherapy Versus Placebo Combined With Postoperative Adjuvant Chemotherapy in Patients With Gastric or Gastroesophageal Junction Adenocarcinoma After Radical Gastrectomy |
| NCT05111626 | A Phase 1b/3 Study of Bemarituzumab Plus Chemotherapy and Nivolumab Versus Chemotherapy and Nivolumab Alone in Subjects With Previously Untreated Advanced Gastric and Gastroesophageal Junction Cancer With FGFR2b Overexpression |
| NCT05144854 | A Randomized, Multicenter, Open-label, Phase III Study to Compare the Efficacy and Safety of ONO-4538 in Combination With Ipilimumab, Fluoropyrimidine-based and Platinum-based Chemotherapy (Hereinafter Referred to as "Chemotherapy") Versus Chemotherapy in Chemotherapy-naïve Participants With Human Epidermal Growth Factor Receptor 2 (HER2)-Negative Unresectable Advanced or Recurrent Gastric Cancer (Including Esophagogastric Junction Cancer) |
| NCT05052801 | A Randomized, Multi-center, Double-blind, Placebo-controlled Phase 3 Study of Bemarituzumab Plus Chemotherapy Versus Placebo Plus Chemotherapy in Subjects With Previously Untreated Advanced Gastric or Gastroesophageal Junction Cancer With FGFR2b Overexpression |
| EUCTR2021-000296-36-PT | A Randomized, Multicenter, Phase 3 Study of Zanidatamab in Combination with Chemotherapy with or without Tislelizumab in Subjects with HER2-positive Unresectable Locally Advanced or Metastatic Gastroesophageal Adenocarcinoma (GEA) - HERIZON-GEA-01 |
| NCT05008783 | A Randomized, Double-blind, Multicenter, Phase III Study Comparing the Efficacy and Safety of AK104 Plus Oxaliplatin and Capecitabine (XELOX) Versus Placebo Plus XELOX as First-line Treatment for Locally Advanced Unresectable or G/GEJ Adenocarcinoma |
| NL-OMON55260 | A Randomized, Double-blind, Placebo-controlled, Phase III Study of Neoadjuvant-Adjuvant Durvalumab and FLOT Chemotherapy Followed by Adjuvant Durvalumab or Placebo in Patients with Resectable Gastric and Gastroesophageal Cancer (GC/GEJC) (MATTERHORN) - MATTERHORN |
| NCT05002127 | A Phase 2/3 Study of Evorpacept (ALX148) in Patients With Advanced HER2-Overexpressing Gastric/Gastroesophageal Junction Adenocarcinoma (ASPEN-06) |
| ChiCTR2100048255 | A randomized, open-label phase III clinical study on the efficacy and safety of oxaliplatin combined with capecitabine versus docetaxel combined with Seggio in the treatment of stage IIIc gastric cancer with postoperative adjuvant chemotherapy |
| NCT04950322 | A Randomized, Double-Blind, Multi-Center, Phase III Clinical Study of SHR-1701 Plus Chemotherapy Versus Placebo Plus Chemotherapy as Treatment in Patients With Previously Untreated, Advanced or Metastatic Gastric or Gastroesophageal Junction Cancer |
| NCT04787354 | A Randomized Phase 3 Clinical Trial Investigating Optimal Duration of Oxaliplatin Administration in Postoperative XELOX (Oxaliplatin + Capecitabine) Adjuvant Chemotherapy for the Patients With Stage II/III Gastric Cancer |
| NCT04714190 | Randomized, Controlled, Multicenter Phase III Clinical Study Evaluating the Efficacy and Safety of RC48-ADC for the Treatment of Locally Advanced or Metastatic Gastric Cancer With HER2-overexpression |
| NCT04704934 | A Phase 3, Multicenter, 2-Arm Randomized, Open-Label Study of Trastuzumab Deruxtecan in Subjects With HER2-Positive Metastatic and/or Unresectable Gastric or Gastro-Esophageal Junction (GEJ) Adenocarcinoma Subjects Who Have Progressed on or After a Trastuzumab-Containing Regimen (DESTINY-Gastric04) |
| CTRI/2021/01/030183 | Neoadjuvant chemotherapy followed by Surgery versus Surgery followed by adjuvant chemotherapy for resectable Gastric adenocarcinoma- 2x2 Factorial Phase 3-Multicentric Randomized Controlled trial - NAGA |
| NCT04675983 | A Randomized, Multicenter, Phase 3 Study to Evaluate the Efficacy and Safety of Sintilimab Combined With Ramucirumab as Compared to Chemotherapy for the First-line Treatment of Unresectable Locally Advanced or Metastatic Gastric or Gastroesophageal Junction Adenocarcinoma (ORIENT-106) |
| EUCTR2020-001990-53-FR | A Phase 3, Randomized Study to Evaluate the Efficacy and Safety of Pembrolizumab (MK-3475) plus Lenvatinib (E7080/MK-7902) plus Chemotherapy Compared with Standard of Care Therapy as First-line Intervention in Participants with Advanced/Metastatic HER2 Negative Gastric/Gastroesophageal Junction Adenocarcinoma (LEAP-015) |
| NCT04597294 | A Randomized, Multicenter Clinical Trial Comparing the Combination of Perioperative FLOT Chemotherapy and Preoperative Laparoscopic Hyperthermic Intraperitoneal Chemotherapy (HIPEC) Plus Gastrectomy to Perioperative FLOT Chemotherapy and Gastrectomy Alone in Patients With Advanced Gastric Cancer at High Risk of Peritoneal Recurrence |
| JPRN-jRCT2031200087 | Randomized phase III trial comparing combined systemic and intraperitoneal chemotherapy versus systemic chemotherapy as adjuvant or perioperative chemotherapy in patients with type 4 advanced gastric cancer - PHOENIX-GC2 trial |
| ChiCTR2000035665 | A Phase III, Prospective, Multicenter, Randomized, Controlled Trial of Neoadjuvant Transcatheter Arterial Chemoembolization (TACE) plus PD-1 Antibody (Tislelizumab) in the Locally Advanced Stomach Adenocarcinoma |
| NCT04499924 | A Randomized, Double-blind, Placebo-controlled, Active Comparator Phase 2/3 Study of Tucatinib in Combination With Trastuzumab, Ramucirumab, and Paclitaxel in Subjects With Previously Treated, Locally-advanced Unresectable or Metastatic HER2+ Gastric or Gastroesophageal Junction Adenocarcinoma (GEC) |
| NCT04486651 | A Randomized, Double-blinded, Multicenter, Phase III Clinical Study of HX008 (Recombinant Humanized Anti-PD-1 Monoclonal Antibody Injection) Plus Irinotecan Versus Placebo Plus Irinotecan as Second-line Treatment in Advanced Gastric Cancer |
| NCT04483076 | A Prospective, Multicenter, Randomized, Controlled Phase III Study Evaluating Different Cycles of Oxaliplatin Combined With S-1 (SOX) as Neoadjuvant Chemotherapy for Patients With Locally Advanced Gastric Cancer: RESONANCE-II Trial |
| ChiCTR2000034770 | Adjuvant chemotherapy of docetaxel plus capecitabine versus oxalipatin plus capecitabine for gastric cancer after D2 gastrectomy: an open-label, phase III randomised controlled trial |
| NCT04435652 | A Study of QL1604 Plus Nab-paclitaxel Versus Paclitaxel for Participants With Advanced Gastric or Gastroesophageal Junction Adenocarcinoma That Progressed After Therapy With Platinum and Fluoropyrimidine |
| NCT04385550 | A Randomized, Open-label, Controlled, Multicenter Phase III Study of Hydrochloride Capsule Combined With AK105 Injection Versus Standard Second-line Chemotherapy for Advanced Gastric and Gastro-oesophageal Junction Adenocarcinoma |
| NCT04384601 | Efficacy Study of FLOT Versus SOX Regimen as Neoadjuvant Chemotherapy for Patients With Locally Advanced Gastric Cancer: A Phase 3 Multi-center Randomized Controlled Trial |
| EUCTR2017-003832-35-DE | Preventive HIPEC in combination with perioperative FLOT versus FLOT alone for resectable diffuse type gastric and gastroesophageal junction Type II/III adenocarcinoma –  The phase III “PREVENT” trial of the AIO /CAOGI /ACO |
| NCT04375605 | RACE-trial: Neoadjuvant Radiochemotherapy Versus Chemotherapy for Patients With Locally Advanced, Potentially Resectable Adenocarcinoma of the Gastroesophageal Junction (GEJ) A Randomized Phase III Joint Study of the AIO, ARO and DGAV |
| NCT04393584 | Perioperative Chemotherapy With FOLFIRINOX Regimen or FLOT Regimen for Resectable Gastric or Esophagogastric Junction Adenocarcinoma (Type II-III): Open-label Randomized Phase 2/3 Trial |
| NCT04358354 | Irinotecan Plus Oxaliplatin, 5-fluorouracil and Leucovorin as First-line Treatment for Metastatic Gastric Cancer |
| NCT04342910 | A Study of Camrelizumab (SHR-1210) Combined With Apatinib Versus Paclitaxel or Irinotecan in Participants With Advanced Gastric/Gastroesophageal Junction Adenocarcinoma Progressed After First-line Chemotherapy |
| NCT04351867 | The Efficacy and Safety of Postoperative Chemotherapy With Docetaxel Plus Oxaliplatin and Capecitabine Versus Oxaliplatin Plus Capecitabine for Postoperative Pathological Stage IIIB/IIIC Gastric Adenocarcinoma: a Randomised, Phase 3 Trial |
| NCT04208347 | Perioperative Treatment of Combined SOX With Apatinib and Camrelizumab for Resectable Locally Advanced Gastric or Esophagogastric Junction Adenocarcinoma: A Multicenter, Randomized Phase II-III Trial |
| NCT04135781 | Adjuvant Nab-paclitaxel Plus S-1 Versus Capecitabine Plus Oxaliplatin for Patients With Stage III Gastric Cancer After D2 Gastrectomy : a Randomised,Open-label, Phase III Study |
| NCT04139135 | A Randomized, Double-blinded, Multicenter, Phase III Clinical Study of HLX10 (Recombinant Humanized Anti-PD-1 Monoclonal Antibody Injection) Combined With Chemotherapy Versus Placebo Combined With Chemotherapy for Neoadjuvant/Adjuvant Treatment of Gastric Cancer |
| NCT04082364 | A Phase 2/3 Trial to Evaluate Margetuximab in Combination With INCMGA00012 and Chemotherapy or MGD013 and Chemotherapy in Patients With Metastatic or Locally Advanced, Treatment-naïve, HER2-Positive Gastric or Gastroesophageal Junction Cancer |
| ChiCTR1900024552 | Combined neoadjuvant chemotherapy (NAC) and laparoscopic intraperitoneal hyperthermic chemotherapy (L-HIPEC) followed by R0 gastrectomy for locally advanced gastric cancer with serosal invasion (cT4-LAGC): A multicentric randomized controlled trial |
| NCT03986385 | Prospective, Randomized, Controlled, Multicenter, Phase III Study of Apatinib Plus Concurrent Neoadjuvant Chemoradiotherapy for Siewert II ,III of Locally Advanced HER-2 Negative Adenocarcinoma at Gastroesophageal Junction |
| NCT03973008 | Phase III, Randomized, Open-label Study of Adjuvant Chemotherapy Combined With Chemoradiotherapy Versus Adujvant Chemotherapy After Standard D2 Resection for Locally Advanced Proximal Gastric Adenocarcinoma |
| NCT03961867 | S-1 Plus Docetaxel(DS) Versus S-1 Plus Oxaliplatin(SOX) as Postoperative Therapy for Stage II / III Gastric Cancer, a Randomized Controlled Trial |
| ChiCTR1900023293 | A prospective multicenter, randomized, controlled phase III study of oxaliplatin combined with S-1(SOX) neoadjuvant chemotherapy for different cycles in patients with locally advanced gastric adenocarcinoma |
| NCT03941561 | Compare S-1 for 9 Months to 1 Year as Adjuvant Chemotherapy in Stage II Gastric Cancer (SMAC) |
| JPRN-jRCTs041180146 | A Phase III Study of Docetaxel plus S-1 versus S-1 Alone in the Treatment of Curatively Resected Stage III Gastric Cancer(JACCRO GC-07(START-2)) |
| JPRN-jRCTs031180350 | JCOG1509: Phase III trial to evaluate the efficacy of neoadjuvant chemotherapy with S-1 plus oxaliplatin followed by D2 gastrectomy with adjuvant S-1 in locally advanced gastric cancer. - NAGISA trial |
| NCT03889626 | A Phase III Study of Comparing the Maintenance Treatment of Apatinib, Capecitabine and Observation After First-line Therapy in Advanced Gastric Cancer |
| JPRN-jRCTs031180255 | JCOG1507: A phase III trial to confirm S-1 adjuvant chemotherapy for pathological stage II/III vulnerable elderly gastric cancer patients who underwent gastric resection - BIRDIE |
| NCT03813784 | A Randomized, Multicenter, Open-Label, Phase 3 Study Comparing the Efficacy and Safety of SHR-1210 Plus Capecitabine and Oxaliplatin Sequenced by Apatinib With or Without SHR-1210 Versus Capecitabine and Oxaliplatin in Subjects With Previously Untreated Advanced or Metastatic Gastric or Gastroesophageal Junction Cancer |
| NCT03817268 | Capecitabine or Observation for Patients With pT1N+M0 or pT2-3N0M0 Gastric Adenocarcinoma Undergoing R0 Resection (CAPOGA): A Large Multicenter Phase III Randomized Controlled Trial |
| NCT03802591 | A Multi-Center, Double-Blind, Randomized, Phase III Study of CS1001 in Combination With CAPOX Chemotherapy Compared to Placebo in Combination With CAPOX Chemotherapy in Subjects With Unresectable Locally Advanced or Metastatic GC or GEJ Adenocarcinoma |
| NCT03801668 | A Multi-center, Open-label, Randomized Controlled Study of Albumin-bound Paclitaxel Plus S-1 Versus Oxaliplatin Plus S-1 (SOX) as First-line Treatment in Advanced or Recurrent Gastric Adenocarcinoma |
| NCT03788226 | A Randomized Phase III Study Comparing POF (Paclitaxel/Oxaliplatin/Leucovorin/5-FU) With SOX/CAPOX/FOLFOX as a Postoperative Adjuvant Chemotherapy for Curatively Resected Stage III Gastric Cancer |
| NCT03745170 | Efficacy and Safety Evaluation of Sintilimab or Placebo in Combination With XELOX as First Line Treatment in Patients With Gastric Cancer |
| NCT03777657 | A Randomized, Double-Blind, Placebo-Controlled, Phase 3 Clinical Study Comparing the Efficacy and Safety of Tislelizumab (BGB-A317) Plus Platinum and Fluoropyrimidine Versus Placebo Plus Platinum and Fluoropyrimidine as First-Line Treatment in Patients With Locally Advanced Unresectable or Metastatic Gastric or Gastroesophageal Junction Adenocarcinoma |
| NCT03680261 | Phase III Multicenter Randomized Controlled Trial of Adjuvant Chemoradiotherapy vs Chemotherapy for Radical Resected Advanced Gastric Carcinoma Concurrent With Lymph Node Metastasis and Lymphovascular Invasion |
| NCT03675737 | A Phase 3, Randomized, Double-blind Clinical Study of Pembrolizumab (MK-3475) Plus Chemotherapy Versus Placebo Plus Chemotherapy as First-line Treatment in Participants With HER2 Negative, Previously Untreated, Unresectable or Metastatic Gastric Orgastroesophageal Junction Adenocarcinoma (KEYNOTE-859) |
| NCT03691454 | A Randomized, Multicenter, Controlled, Adaptive II/III Study to Compare Neoadjuvant Chemotherapy of Docetaxel,Oxaliplatin Combined With S-1(DOS) Versus Oxaliplatin Combined With S-1(SOX)in Locally Advanced Gastric Adenocarcinoma (RESOLVE-2 Study) |
| NL-OMON46592 | A Randomized, Active-Controlled, Blinded, Phase III Clinical Trial of BMS-986213 (Fixed Dose Combination of Relatlimab [anti-LAG-3] and Nivolumab) in Combination with Chemotherapy versus Placebo in Combination with Chemotherapy as First-Line Treatment in Participants with Unresectable, Locally Advanced or Metastatic LAG-3 Postive Gastric or Gastroesophageal Junction Adenocarcinoma - CA224-051 |
| NCT03653507 | A Phase 3, Global, Multi-Center, Double-Blind, Randomized, Efficacy Study of Zolbetuximab (IMAB362) Plus CAPOX Compared With Placebo Plus CAPOX as First-line Treatment of Subjects With Claudin (CLDN) 18.2-Positive, HER2-Negative, Locally Advanced Unresectable or Metastatic Gastric or Gastroesophageal Junction (GEJ) Adenocarcinoma |
| NCT03604991 | A Phase II/III Study of Peri-Operative Nivolumab and Ipilimumab in Patients With Locoregional Esophageal and Gastroesophageal Junction Adenocarcinoma |
| EUCTR2018-000224-34-ES | A Phase III, Randomized, Double-blind Trial Comparing Trastuzumab Plus Chemotherapy and Pembrolizumab With Trastuzumab Plus Chemotherapy and Placebo as First-line Treatment in Participants With HER2 Positive Advanced Gastric or Gastroesophageal Junction Adenocarcinoma (KEYNOTE 811) |
| NCT03504397 | A Phase 3, Global, Multi-Center, Double-Blind, Randomized, Efficacy Study of Zolbetuximab (IMAB362) Plus mFOLFOX6 Compared With Placebo Plus mFOLFOX6 as First-line Treatment of Subjects With Claudin (CLDN)18.2-Positive, HER2-Negative, Locally Advanced Unresectable or Metastatic Gastric or Gastroesophageal Junction (GEJ) Adenocarcinoma |
| NCT03475615 | A Randomized Study of the Effect of First-line Intraperitoneal Paclitaxel in Combination With SOX Versus SOX Alone in Gastric Cancer Patients With Malignant Ascites |
| ISRCTN12206108 | Efficacy and safety of oxaliplatin plus S-1 or capecitabine as neoadjuvant or adjuvant chemotherapy for locally advanced gastric cancer with D2 lymphadenectomy: a phase II-III randomized trial |
| NCT03399110 | XELOX for 4 Months Versus 6 Months as Adjuvant Chemotherapy in Gastric Cancer After D2 Resection (LOMAC) |
| NCT03399253 | A Prospective,Multicentral,Open-label,Randomized,Controlled,Phase III Clinical Trial of Chemotherapy Alone Versus D2 Gastrectomy and Metastasectomy Plus Chemotherapy for Distal Gastric Cancer With One Non-curable Factor |
| NCT03598348 | Maintenance Treatment With Capecitabine Plus Apatinib vs. Apatinib and Observation After First-line XELOX/SOX Chemotherapy for Patients With Advanced Gastric Cancer: a Multicenter, Randomized, Controlled Trial |
| CTRI/2017/11/010651 | Comparison of efficacy of aspirin plus Epirubicin,Oxaliplatin,Capecitabine(EOX)vs Epirubicin,Oxaliplatin,Capecitabine(EOX)alone in patients with locally advanced and metastatic gastric cancer-a randomised pilot trial |
| NCT03355612 | A Randomized, Multicenter, Controlled Study of XELOX (Oxaliplatin With Capecitabine) Combined With Apatinib Versus XELOX as Post-operative Chemotherapy in Locally Advanced Gastric Signet Ring Carcinoma With D2 Dissection. |
| NCT03223740 | A Phase III Trial of Preoperative or Postoperative Chemoradiation Therapy for Potentially Resectable Adenocarcinoma of Stomach Cancer |
| NCT03223376 | A Phase III Study to Evaluate the Efficacy and Safety of Fruquintinib in Combination With Paclitaxel Versus Paclitaxel Alone in Second Line Gastric Cancer |
| NCT03221426 | A Phase III, Randomized, Double-Blind, Clinical Trial of Pembrolizumab (MK-3475) Plus Chemotherapy (XP or FP) Versus Placebo Plus Chemotherapy (XP or FP) as Neoadjuvant/Adjuvant Treatment for Subjects With Gastric and Gastroesophageal Junction (GEJ) Adenocarcinoma (KEYNOTE-585) |
| NCT03179579 | A Phase III Study of Hyperthermic Intraperitoneal Chemotherapy (HIPEC) Combined With Systemic Chemotherapy And Cytoreductive Surgery (CRS) in the Treatment of Peritoneal Carcinomatosis From Gastric Cancer |
| NCT03130790 | A Two-Part Phase 2/ 3 Multicentre, Double-Blind, Randomized, Placebo Controlled Study of Varlitinib Plus mFOLFOX6 Verses Placebo Plus mFOLFOX6 In Subjects With HER1/ HER2 Co Expressing Advanced or Metastatic Gastric Cancer Without Prior Exposure to Systemic Therapy |
| NCT03081143 | Ramucirumab Plus Irinotecan / Leucovorin / 5-FU Versus Ramucirumab Plus Paclitaxel in Patients With Advanced or Metastatic Adenocarcinoma of the Stomach or Gastroesophageal Junction, Who Failed One Prior Line of Palliative Chemotherapy - The Phase II/III RAMIRIS Study |
| NCT03067792 | FOLFIRI Versus Docetaxel and Cisplatin as a Second-line Chemotherapy After Failure of First-line Chemotherapy in Advanced Gastric Cancer |
| NCT03061058 | An Open-label, Randomized, Phase III, Multicenter Clinical Trial Comparing the Efficacy and Safety of Individualized Intraperitoneal and System Chemotherapy Versus System Chemotherapy as First-line Chemotherapy for Advanced Gastric Cancer |
| NCT03042169 | Surgical Resection Plus Chemotherapy Versus Chemotherapy Alone in Oligometastatic Stage IV Gastric Cancer - a Multicenter, Prospective, Open-labeled, Two-armed, Randomized, Controlled Phase III Trial |
| NCT03042611 | A Prospective, Randomized, Double-Blinded, Placebo-Controlled, Multinational, Multicenter, Parallel-group, Phase III Study to Evaluate the Efficacy and Safety of Apatinib Plus Best Supportive Care (BSC) Compared to Placebo Plus BSC in Patients With Advanced or Metastatic Gastric Cancer |
| JPRN-UMIN000025742 | A phase III trial to confirm S-1 adjuvant chemotherapy for pathological stage II/III vulnerable elderly gastric cancer patients who underwent gastric resection (JCOG1507, BIRDIE) - A phase III trial to confirm S-1 adjuvant chemotherapy for pathological stage II/III vulnerable elderly gastric cancer patients who underwent gastric resection (JCOG1507, BIRDIE) |
| NCT03019588 | A Phase III, Randomized, Open-label Clinical Trial of Pembrolizumab (MK-3475) Versus Paclitaxel in Asian Subjects With Advanced Gastric or Gastroesophageal Junction Adenocarcinoma Who Progressed After First-Line Therapy With Platinum and Fluoropyrimidine |
| NCT03013010 | A Randomized, Controlled, Multicenter Study to Compare Preoperative Radiochemotherapy With Preoperative Chemotherapy in Patients With Locally Advanced Gastric or Esophagogastric Junction Adenocarcinoma (PREACT Study) |
| NCT03006432 | ESSAI DE PHASE III RANDOMISE EVALUANT LE FOLFOX AVEC OU SANS DOCETAXEL (TFOX) EN 1ère LIGNE DE CHIMIOTHERAPIE DES ADENOCARCINOMES OESO-GASTRIQUES LOCALEMENT AVANCES OU METASTATIQUES |
| NCT02934464 | Assessment of Ramucirumab Plus Paclitaxel as Switch MANteInance Versus Continuation of First-line Chemotherapy in Patients With Advanced HER-2 Negative Gastric or Gastroesophageal Junction Cancers: the ARMANI Phase III Trial |
| JPRN-UMIN000024065 | Phase III trial to evaluate the efficacy of neoadjuvant chemotherapy with S-1 plus oxaliplatin followed by D2 gastrectomy with adjuvant S-1 in locally advanced gastric cancer.(JCOG1509, NAGISA trial) - Phase III trial to evaluate the efficacy of neoadjuvant chemotherapy with S-1 plus oxaliplatin followed by D2 gastrectomy with adjuvant S-1 in locally advanced gastric cancer.(JCOG1509, NAGISA trial) |
| NCT02898077 | A Randomized, Multicenter, Double-Blind, Placebo-Controlled, Phase 3 Study of Weekly Paclitaxel With or Without Ramucirumab (IMC-1121B) in Patients With Advanced Gastric or Gastroesophageal Junction Adenocarcinoma, Refractory to or Progressive After First-Line Therapy With Platinum and Fluoropyrimidine |
| EUCTR2016-001018-76-ES | A Randomized, Multicenter, Open-Label, Phase 3 Study of Nivolumab Plus Ipilimumab versus Oxaliplatin plus Fluoropyrimidine in Subjects with Previously Untreated Advanced or Metastatic Gastric or Gastroesophageal Junction Cancer (CheckMate 649: CHECKpoint pathway and nivoluMab clinical Trial Evaluation 649) - CheckMate 649 |
| NCT02867839 | A Randomized, Multicentral, Controlled, Phase III Study to Compare Adjuvant Chemotherapy With S-1 Plus Oxaliplatin to S-1 Alone After Curative Distal Gastrectomy in Locally Advanced Gastric Cancer |
| JPRN-UMIN000023065 | An intergroup phase III trial of Ramucirumab plus Irinotecan in third or more line Beyond progression after Ramucirumab for Advanced Gastric cancer. - RINDBeRG trial |
| JPRN-jRCT2080223213 | A multicenter, double-blind, randomized study in patients with gastric cancer undergoing postoperative adjuvant chemotherapy (ONO-4538-38/BMS CA209844) |
| ACTRN12616000420448 | A Randomised Phase III Double-Blind Placebo-Controlled Study to determine if regorafenib improves overall survival in refractory Advanced Gastro-Oesophageal Cancer (AGOC) |
| NCT02746796 | ONO-4538 Phase II/III Study A Multicenter, Randomized Study in Patients With Unresectable Advanced or Recurrent Gastric Cancer |
| NCT02648841 | A Randomized Phase III Study of Adjuvant Chemotherapy With or Without Chemo-radiotherapy in Patients With Local Advanced Gastric Cancer After D2 Resection |
| NCT02625623 | A Phase III Open-label, Multicenter Trial of Avelumab (MSB0010718C) as a Third-line Treatment of Unresectable, Recurrent, or Metastatic Gastric or Gastroesophageal Junction Adenocarcinoma |
| NCT02625610 | A Phase III Open-label, Multicenter Trial of Maintenance Therapy With Avelumab (MSB0010718C) Versus Continuation of First-line Chemotherapy in Subjects With Unresectable, Locally Advanced or Metastatic, Adenocarcinoma of the Stomach, or of the Gastro-esophageal Junction |
| NCT02581462 | FLOT vs. FLOT/Herceptin/Pertuzumab for Perioperative Therapy of Adenocarcinoma of the Stomach and Gastroesophageal Junction Expressing HER-2. A Phase II/III Trial of the AIO. |
| NCT02578368 | Effect of Chemotherapy Alone vs. Chemotherapy Followed by Surgical Resection on Survival and Quality of Life in Patients With Limited-metastatic Adenocarcinoma of the Stomach or Esophagogastric Junction - A Phase III Trial of Arbeitsgemeinschaft Internistische Onkologie/Chirurgische Arbeitsgemeinschaft Onkologie/Chirurgische Arbeitsgemeinschaft für Den Oberen Gastrointestinaltrakt (AIO/CAO-V/CAOGI) |
| NCT02545504 | A Phase 3 Randomized, Double-Blind, Placebo-Controlled Study to Evaluate the Efficacy and Safety of GS-5745 Combined With mFOLFOX6 as First Line Treatment in Patients With Advanced Gastric or Gastroesophageal Junction Adenocarcinoma |
| NCT02512380 | Neoadjuvant S1, Oxaliplatin, and Docetaxel (SLOT) Versus S1, Oxaliplatin(SOX) in Patients With Locally Advanced, Resectable Gastric/Esophagogastric Junction (EGJ) Cancer |
| NCT02509806 | Apatinib as Maintenance Therapy After First-line Chemotherapy(DC for 4 Cycles) in Postoperative Recurrence/​Metastasis Progressive Gastric Cancer |
| NCT02510469 | Apatinib as Maintenance Therapy After Adjuvant Chemotherapy in Progressive Gastric Cancer With Positive Exfoliative Cancer Cells |
| NCT02537171 | Apatinib as Maintenance Therapy After First Line Treatment in Locally Advanced or Metastatic Gastric Cancer: A Randomized, Parallel, Controlled Study |
| NCT02500043 | Randomized, Double-blind, Phase 3 Study Evaluating TAS-102 Plus Best Supportive Care (BSC) Versus Placebo Plus BSC in Patients With Metastatic Gastric Cancer Refractory to Standard Treatments |
| NCT02817425 | A Efficacy and Tolerability Study of Uracil/Ftorafur/Leucovorin Combined With Oxaliplatin (TEGAFOX) Sequential S-1 or SOX Sequential S-1 and S-1 Monotherapy in the Treatment of Adjuvant Chemotherapy for Gastric Cancer |
| NCT02461407 | A Randomized, Double-blind, Placebo-controlled, Multicenter Clinical Trial to Compare the Efficacy and Safety of Anlotinib Versus Placebo in Patients With Gastric Cancer(ALTER0503) |
| NCT02442362 | Paclitaxel/Oxaliplatin/Fluorouracil (TOF) Regimen Versus S-1/Oxaliplatin (SOX) Regimen Metastatic Gastric Cancer Patients |
| NCT02445209 | Comparison of Efficacy and Frequency of Adverse Events of 1st Line Palliative Chemotherapy EOX and mDCF Regimens in Advanced HER2-negative Gastric Carcinoma |
| NCT02409199 | A Randomized, Multicenter Study To Evaluate The Efficacy And Safety Of Apatinib Versus Docetaxel In Patients With Previously Treated Locally Advanced Or Metastatic Gastric Cancer, Including Adenocarcinoma Of The Gastroesophageal Junction |
| NCT02395640 | The Comparison of XELOX and EOX in the First-line Treatment of Advanced Gastric Cancer: An Open-label, Multi-center, Prospective and Randomised Study |
| EUCTR2014-005241-45-FI | A Phase III, Randomized, Open-label Clinical Trial of Pembrolizumab (MK-3475) versus Paclitaxel in Subjects with Advanced Gastric or Gastroesophageal Junction Adenocarcinoma who Progressed after First-Line Therapy with Platinum and Fluoropyrimidine - Pembrolizumab (MK-3475) vs Paclitaxel in 2L Subjects with Advanced Gastric Adenocarcinoma |
| NCT02322593 | An Open-label Randomized Multi-center Phase III Study of TAS-118 Plus Oxaliplatin Versus S-1 Plus Cisplatin as First-line Therapy in Patients With Advanced Gastric Cancer |
| NCT02314117 | A Randomized, Double-Blind, Placebo-Controlled Phase 3 Study of Capecitabine and Cisplatin With or Without Ramucirumab as First-line Therapy in Patients With Metastatic Gastric or Gastroesophageal Junction Adenocarcinoma (RAINFALL) |
| NCT02396498 | S-1 With Cisplatin Intravenous Chemotherapy Versus S-1 Plus Cisplatin Hyperthermic Intraperitoneal Chemotherapy in Stage ? Patients After D2 Gastrectomy:a Randomized Controlled Study |
| NCT02289547 | Randomized Phase 3 Study of Xelox(Capecitabine Plus Oxaliplatin) Followed by Maintenance Capecitabine or Observation in Patients With Advanced Gastric Adenocarcinoma |
| NCT02338518 | Comparison of SEEOX and SOX Chemotherapeutic Regimens in Stage ?B/?C Gastric Cancer Patients |
| NCT02205008 | Prospective Randomized Multicenter Phase III Trial of Intraperitoneal Chemotherapy and Systemic Chemotherapy Versus Systemic Chemotherapy After Curative Resection of Serosa-positive Gastric Cancer |
| NCT02193594 | Phase ?/? Study of Preoperative Concurrent Chemoradiotherapy for Locally Advanced Gastroesophageal Junction or Upper Gastric Adenocarcinoma |
| NCT02178956 | A Phase III Clinical Trial of BBI608 Plus Weekly Paclitaxel vs. Placebo Plus Weekly Paclitaxel in Adult Patients With Advanced, Previously Treated Gastric and Gastro-Esophageal Junction Adenocarcinoma |
| NCT02158988 | Prospective Multicenter Phase III Trial Using CRS With / Without HIPEC After Preoperative Chemotherapy in Patients With Peritoneal Carcinomatosis of Gastric Cancer Incl. Adenocarcinoma of the Esophagogastric Junction |
| NCT02137343 | A Phase 3, Multicenter, Randomized, Double-Blind, Placebo Controlled Study of Rilotumumab (AMG 102) With Cisplatin and Capecitabine (CX) as First-line Therapy in Advanced MET-Positive Gastric or Gastroesophageal Junction Adenocarcinoma |
| NCT02114359 | Comparison of Efficacy and Tolerance Between Combination Therapy and Monotherapy as a First Line Chemotherapy in Elderly Patient With Advanced Gastric Cancer; Multicenter Randomized Phase 3 Study |
| JPRN-jRCT2080222339 | A Randomized, Double-blinded, Placebo Controlled, Multicentre Phase 3 Study to Assess the Efficacy and Safety of Olaparib (AZD2281) in Combination With Paclitaxel, Compared to Placebo in Combination With Paclitaxel, in Asian Patients With Advanced Gastric Cancer (Including the Gastro-oesophageal Junction) Who Have Progressed Following First Line Therapy |
| NCT02008422 | A Randomized, Open and Control Clinical Study of Endostar Injection Concomitant With SOX Protocols in Treating Advanced Gastric Cancer |
| NCT01935778 | A Phase 3, Open-Label, Randomized Study to Compare Adjuvant Chemotherapy of Docetaxel/Capecitabine/Oxaliplatin Versus Capecitabine/Oxaliplatin in Advanced Gastric Cancer Patients at Stage IIIB and IV (M0) (Based on AJCC Ed. 6) Who Received Radical Resection(KCSG ST15-08) |
| NCT02076594 | A Randomized Phase III Study Of Low-Docetaxel Oxaliplatin, Capecitabine (Low-Tox) Vs Epirubicin, Oxaliplatin And Capecitabine (Eox) In Patients With Locally Advanced Unresectable Or Metastatic Gastric Cancer |
| NCT01917552 | Phase III Study of Adjuvant Capecitabine vs Observation Alone in Curatively Resected Stage IB (by AJCC 6th Edition) Gastric Cancer(KCSG ST14-05) |
| JPRN-UMIN000010949 | Randomized phase II/III study of 5-fluorouracil /l-leucovorin vs. 5-fluorouracil /l-leucovorin plus paclitaxel in gastric cancer with severe peritoneal metastasis (JCOG1108/WJOG7312G, abbreviation:GC_FL/FLTAX_P2/3) - Phase II/III study of 5-FU/l-LV vs. 5-FU/l-LV+PTX in gastric cancer with severe peritoneal metastasis (FL/FLTAX trial) |
| CTRI/2013/05/003708 | Randomized controlled trial of perioperative versus postoperative chemotherapy in resectable carcinoma stomach - SIOG-STO-1 |
| NCT01839773 | A Randomized, Open, Multicenter Phase 3 Study for Efficacy and Safety Assessment of DHP107 (Oral Paclitaxel) vs. Taxol® in Patients With Metastatic or Recurrent Gastric Cancer After Failure of First-line Chemotherapy With Fluoropyrimidine +/- Platinum |
| NCT01824459 | A Randomized, Open, Multi-center, Phase III Study of S-1+Oxaliplatin vs.S-1+Cisplatin First-line Treatment in Advanced or Recurrent Non-intestinal Type Gastric Adenocarcinoma or Gastroesophageal Junction Adenocarcinoma Patients |
| NCT01815853 | An Randomized, Multicenter, Open-label, Phase III Trial Comparing Neoadjuvant Chemoradiotherapy Versus Chemotherapy in Patients With Locally Advanced Gastric Adenocarcinoma |
| NCT01813253 | A Randomized, Open-label, Japan-Korea-Taiwan Collaborative Phase 3 Study to Compare the Efficacy of Nimotuzumab and Irinotecan Combination Therapy Versus Irinotecan Monotherapy as Second Line Treatment in Subjects With Advanced or Recurrent Gastric and Gastroesophageal Junction Cancer |
| ChiCTR-TRC-13003139 | Paclitaxel plus S-1 (TS) versus oxaliplatin and S-1(SOX) for treatment of advanced diffuse-type gastric cancer: a multicenter, randomised ,controlled trial |
| NCT01795027 | A Prospective,Multicentral,Open-label,Randomized,Controlled,Phase III Clinical Trial to Compare S-1 Plus Oxaliplatin to S-1 as Adjuvant Chemotherapy After D2 Resection in Patients With Gastric Cancer |
| NCT01787539 | The Role of Postoperative Chemotherapy Cycles in the Combined Modality Therapy of Gastric Cancer With Perioperative Chemotherapy and Surgery in Pathological Responders |
| JPRN-jRCT2080222013 | Randomized, phase III, comparative study of every-3-weeks dosing, weekly dosing of ABI-007, and weekly dosing of the conventional paclitaxel for patients with unresectable/recurrent gastric cancer refractory to prior chemotherapy containing fluoropyrimidines. |
| NCT01774786 | A Double-blind, Placebo-controlled, Randomized, Multicenter Phase III Study Evaluating the Efficacy and Safety of Pertuzumab in Combination With Trastuzumab and Chemotherapy in Patients With HER2-positive Metastatic Gastroesophageal Junction and Gastric Cancer |
| NCT01761461 | Phase III Randomized Trial of Adjuvant Chemotherapy With S-1 vs S-1/​Oxaliplatin ± Radiotherapy for Completely Resected Gastric Adenocarcinoma : The ARTIST II Trial (ARTIST-II) |
| ChiCTR-TRC-12002919 | A randomized phase III trial of concurrent chemoradiotherapy consisting of S-1 and oxaliplatin (SOX) in gastric cancer with radical D2 lymph node dissection |
| NCT01748851 | A Phase III Trial to Evaluate the Efficacy and Safety of the Combination Therapy of Capecitabine and Oxaliplatin (XELOX) in Comparison to the Combination Therapy of Fluorouracil/Folinic Acid and Oxaliplatin (FOLFOX) in Patients With AGC |
| NCT01697072 | A Phase 3, Multicenter, Randomized, Double-Blind, Placebo Controlled Study of Rilotumumab (AMG102) With Epirubicin, Cisplatin, and Capecitabine (ECX) as First-line Therapy in Advanced MET-Positive Gastric or Gastroesophageal Junction Adenocarcinoma |
| NCT01671449 | Phase III Trial of S-1 and Cisplatin (3 Weekly) Versus S-1 and Oxaliplatin Combination Chemotherapy for First Line Treatment of Advanced Gastric Cancer |
| NCT01962246 | Phase II Multi-Institutional Randomized Trial of Capecitabine Plus Oxaliplatin With Concurrent Radiotherapy in Patients With Potentially Resectable Adenocarcinoma of Gastroesophageal Cancer |
| NCT01641939 | A Randomized, Multicenter, Adaptive Phase II/III Study To Evaluate The Efficacy And Safety Of Trastuzumab Emtansine (T-DM1) Versus Taxane (Docetaxel Or Paclitaxel) In Patients With Previously Treated Locally Advanced Or Metastatic HER2-Positive Gastric Cancer, Including Adenocarcinoma Of The Gastroesophageal Junction |
| EUCTR2012-001402-23-ES | A RANDOMIZED, PHASE III, MULTICENTER, DOUBLE-BLIND, PLACEBO-CONTROLLED STUDY EVALUATING THE EFFICACY AND SAFETY OF ONARTUZUMAB (MetMAb) IN COMBINATION WITH 5-FLUOROURACIL, FOLINIC ACID, AND OXALIPLATIN (mFOLFOX6) IN PATIENTS WITH METASTATIC HER2-NEGATIVE, MET-POSITIVE GASTROESOPHAGEAL CANCER |
| NCT01583361 | Phase III, Randomized, Multicenter, Controlled Evaluation of S-1 and Oxaliplatin as Neoadjuvant Chemotherapy for Advanced Gastric Cancer Patients |
| JPRN-UMIN000007652 | Phase III study of cisplatin plus S-1 (CS) compared with docetaxel and cisplatin plus S-1 (DCS) as first-line therapy for advanced gastric cancer(JCOG1013, GC_CS/DCS_P3 ADOPT) - Adding docetaxel to cisplatin plus tegafur-gimeracil-oteracil for advanced gastric cancer(JCOG1013,GC_CS/DCS_P3 ADOPT) |
| JPRN-UMIN000007306 | Optimal period of adjuvant S-1 chemotherapy for pathological stage II gastric cancer patients who underwent D2 gastrectomy(JCOG1104) - Optimal period of adjuvant S-1 chemotherapy for pathological stage II gastric cancer patients who underwent D2 gastrectomy(JCOG1104, OPAS-1 phase III) |
| NCT01534546 | A Randomized, Multicenter, Controlled Phase III Study to Compare Perioperative Chemotherapy of Oxaliplatin Combined With S-1(SOX) Versus SOX or Oxaliplatin With Capecitabine (XELOX) as Post-operative Chemotherapy in Locally Advanced Gastric Adenocarcinoma With D2 Dissection |
| NCT01523015 | Efficacy and Safety Study of the Combined Modality Therapy in Patients With Potentially Resectable, Locally Advanced Adenocarcinoma of the Esophago-gastric Junction With Preoperative Chemo- and Chemoradiation Followed by Surgical Resection |
| NCT01512745 | A Randomized, Double Blinded, Placebo Controlled Multicenter Phase III Study of Apatinib Mesylate Tablets in the Treatment of Advanced or Metastatic Gastric Cancer |
| NCT01515748 | A Phase III, Open-labelled, Randomised Study of Neoadjuvant Docetaxel+Oxaliplatin+S-1 (DOS) + Surgery + Adjuvant S-1 Versus Surgery + Adjuvant S-1 in Patients With Resectable Advanced Gastric Cancer |
| NCT01516944 | Perioperative Tegafur Gimeracil Oteracil Potassium Capsule Plus Oxaliplatin Versus Capecitabine Plus Oxaliplatin in Patients With Localized Advanced Gastric Cancer |
| DRKS00003078 | Prospective multicenter phase III clinical trial using cytoreductive surgery with hyperthermic intraoperative chemotherapy (HIPEC) after preoperative chemotherapy in patients with peritoneal carcinomatosis of gastric cancer incl. adenocarcinoma of the esophagogastreal junction - Gastripec I |
| NCT01468389 | A Randomized Multicenter Phase III Study:Taxanes or Platinum in Combination With Capecitabine Followed by Capecitabine Alone vs.Taxanes or Platinum Combined With Capecitabine in Advanced Adenocarcinoma of the Stomach or Esophagogastric Junction. |
| ChiCTR-TRC-11001560 | A multi-center clinical trail for Tegafur, gimeracil and oteracil potassium capsules combined with Oxaliplatin in treatment of phase II, IIIa gastric cancer |
| EUCTR2011-001526-19-DE | A randomized, open-label, multicenter Phase IIIb study comparing two trastuzumab dosing regimens, each in combination with cisplatin/ capecitabine chemotherapy, as first-line therapy in patients with HER2-positive metastatic gastric or gastro-esophageal junction adenocarcinoma who have not received prior treatment for metastatic disease. |
| JPRN-UMIN000005930 | Phase III study of S-1 plus intravenous and intraperitoneal paclitaxel versus S-1 plus cisplatin for gastric cancer with peritoneal metastasis - PHOENIX-GC trial |
| EUCTR2011-001858-28-IE | Neo-AEGIS (NEOadjuvant trial in Adenocarcinoma of the oEsophagus and oesophagoGastric Junction International Study): Randomised Clinical Trial of neoadjuvant and adjuvant chemotherapy (Investigator’s choice Modified MAGIC or FLOT regimen) vs. neoadjuvant chemoradiation (CROSS protocol) in adenocarcinoma of the oesophagus and oesophago-gastric junction - Neo-AEGIS |
| NCT01283217 | A Randomized, Multicenter, Open-label, Phase III Trial of Docetaxel and S1 (DS) Versus S1 and Cisplatin (SP) in Curatively Resected (D2) Gastric Cancer of Stage IIIB/IV (M0) |
| EUCTR2009-016019-39-DE | AN OPEN-LABEL, MULTICENTER, RANDOMIZED, PHASE 3 STUDY OF S-1 AND CISPLATIN COMPARED WITH 5-FU AND CISPLATIN IN PATIENTS WITH METASTATIC DIFFUSE GASTRIC CANCER PREVIOUSLY UNTREATED WITH CHEMOTHERAPY - DIGEST |
| NCT01248403 | A Randomized, Double-blind, Multi-center Phase III Study Evaluating Paclitaxel With and Without RAD001 in Patients With Gastric Carcinoma Who Have Progressed After Therapy With a Fluoropyrimidine-containing Regimen |
| NCT01224652 | A Randomized, Multicenter Phase III Study to Assess the Efficacy of Paclitaxel Versus Irinotecan in Patients With Recurrent or Metastatic Gastric Cancer Who Progress Following First-line Therapy |
| NCT01206218 | Evaluation of Customized Treatment According to BRCA1 Assessment in Patients With Advanced Gastric Cancer |
| NCT01196390 | A Phase III Trial Evaluating the Addition of Trastuzumab to Trimodality Treatment of HER2-Overexpressing Esophageal Adenocarcinoma |
| EUCTR2010-021052-25-IT | ITACA-S 2 (Intergroup Trial in Adjuvant Chemotherapy for Adenocarcinoma of the Stomach): comparison of the efficacy of a peri-operative versus a post-operative chemotherapy treatment in patients with operable gastric cancer and assessment of the benefit of a post-operative chemo-radiotherapy - ITACA-S 2 |
| NCT01216644 | A Randomized Multicenter Phase II/III Study Comparing 5-FU, Leucovorin, Oxaliplatin and Docetaxel (FLOT) Versus Epirubicin, Cisplatin and 5-FU (ECF) in Patients With Locally Advanced Resectable Adenocarcinoma of the Esophagogastreal Junction or the Stomach |
| NCT01099085 | Placebo-controlled, Double-blinded Phase III Trial of XP (Capecitabine/CDDP) Simvastatin in Advanced Gastric Cancer Patients |
| JPRN-jRCT2080220988 | Randomized phase III study of S-1 plus oxaliplatin compared with S-1 plus cisplatin as first-line therapy for advanced or recurrent gastric cancer |
| CTRI/2009/091/000498 | EGF110656 : A Phase III Study for ErbB2 Positive Advanced or Metastatic Gastric, Esophageal, or Gastroesophageal Junction Adenocarcinoma Treated With Capecitabine Plus Oxaliplatin With or Without Lapatinib |
| NCT01015339 | A Randomized Multicenter Phase III Study Comparing Paclitaxel Plus Capecitabine With Capecitabine Maintenance Treatment or Cisplatin Plus Capecitabine in Metastatic Adenocarcinoma of the Stomach or Esophagogastric Junction |
| NCT00970138 | A Randomized Phase 2/3 Study of Apatinib as Third Line Treatment in Patients With Metastatic Gastric Carcinoma |
| NCT00941655 | Prospective Randomized Trial Comparing Gastrectomy, Metastasectomy Plus Systemic Therapy Versus Systemic Therapy Alone: GYMSSA Trial |
| NCT00917384 | A Phase 3, Randomized, Double-Blinded Study of IMC-1121B and Best Supportive Care (BSC) Versus Placebo and BSC in the Treatment of Metastatic Gastric or Gastroesophageal Junction Adenocarcinoma Following Disease Progression on First-Line Platinum- or Fluoropyrimidine-Containing Combination Therapy |
| NCT00915382 | Phase III Trial of 3-weekly vs. 5-weekly Schedule of S-1 Plus Cisplatin Combination Chemotherapy for First Line Treatment of Advanced Gastric Cancer. |
| NCT00887822 | A Double-Blind, Randomized, Multicenter, Phase III Study of Bevacizumab in Combination With Capecitabine and Cisplatin Versus Placebo in Combination With Capecitabine and Cisplatin, as First-Line Therapy in Patients With Advanced Gastric Cancer. |
| NCT00879333 | A Randomized, Double-blind, Multi-center Phase III Study Comparing Everolimus (RAD001) Plus Best Supportive Care Versus Placebo Plus Best Supportive Care in Patients With Advanced Gastric Cancer After Progression on 1 or 2 Prior Systemic Chemotherapy |
| ACTRN12609000035224 | TOP GEAR: Trial of Preoperative Therapy for Gastric and Esophagogastric Junction Adenocarcinoma  A randomised II/III trial of preoperative chemoradiotherapy versus preoperative chemotherapy for resectable gastric cancer |
| NCT02494583 | A Randomized, Active-Controlled, Partially Blinded, Biomarker Select, Phase III Clinical Trial of Pembrolizumab as Monotherapy and in Combination With Cisplatin+5-Fluorouracil Versus Placebo+Cisplatin+5-Fluorouracil as First-Line Treatment in Subjects With Advanced Gastric or Gastroesophageal Junction (GEJ) Adenocarcinoma |
| NCT02773524 | A Randomised Phase III Double-Blind Placebo-Controlled Study of Regorafenib in Refractory Advanced Gastro-Oesophageal Cancer (AGOC) |
| NCT04879368 | A Randomised Phase III Open Label Study of Regorafenib + Nivolumab vs Standard Chemotherapy in Refractory Advanced Gastro-Oesophageal Cancer (AGOC) |
| ISRCTN44687907 | Alternative chemotherapy for frail or elderly patients with advanced gastric or oesophageal cancer: a randomised controlled trial |

**Additional Table 3.** Variables affecting the odds of the presence of an upper age limit of 85 years or lower in phase 3 randomized controlled trials in gastric cancer (sensitivity analysis replacing the trial’s start date with the registration date)

| **Cancer stage**  Metastatic  Early/LAR  **Registration date**  2009-2016  2017-2024  **Site in North America^3^**  No  Yes    **Site in Asia^3^**  No  Yes  **Site in Europe^3^**  No  Yes  **Chemotherapy**  No  Yes  **Targeted drug**  No  Yes  **Immunotherapy**  No  Yes  **Sample size**  ≤249  250-499  ≥500  **Primary sponsor**  Commercial  Non-commercial | **Adjusted odds ratio**  **(95% CI)**  Referent  1.41 (0.67-2.96)  Referent  1.49 (0.74-2.98)  Referent  0.08 (0.01-0.40)  Referent  2.09 (0.43-12.87)  Referent  0.45 (0.09-2.55)  Referent  0.90 (0.31-2.66)  Referent  1.07 (0.46-2.54)  Referent  1.56 (0.59-4.31)  Referent  1.13 (0.49-2.63)  1.40 (0.60-3.30)  Referent  2.01 (0.80-5.21) | **p**  -  0.36  -  0.26  -  **<0.001**  -  0.36  **-**  0.34  -  0.83  -  0.87  -  0.37  -  0.76  0.43  -  0.13 |
| --- | --- | --- |

**Additional Table 4.** Variables affecting the odds of the presence of an upper age limit of 85 years or lower in phase 3 randomized controlled trials in gastric cancer (sensitivity analysis for variable ‘sample size’)

| **Cancer stage**  Metastatic  Early/LAR  **Start date**  2009-2016  2017-2024  **Site in North America^3^**  No  Yes    **Site in Asia^3^**  No  Yes  **Site in Europe^3^**  No  Yes  **Chemotherapy**  No  Yes  **Targeted drug**  No  Yes  **Immunotherapy**  No  Yes  **Sample size**  ≤499  ≥500  **Primary sponsor**  Commercial  Non-commercial | **Adjusted odds ratio**  **(95% CI)**  Referent  1.36 (0.64-2.89)  Referent  1.62 (0.79-3.35)  Referent  0.09 (0.01-0.41)  Referent  2.19 (0.45-13.52)  Referent  0.46 (0.09-2.61)  Referent  0.89 (0.31-2.66)  Referent  1.02 (0.43-2.42)  Referent  1.44 (0.55-3.96)  Referent  1.32 (0.65-2.73)  Referent  2.06 (0.84-5.24) | **p**  -  0.42  -  0.18  -  **<0.001**  -  0.33  **-**  0.35  -  0.83  -  0.96  -  0.45  -  0.44  -  0.11 |
| --- | --- | --- |

**Additional Table 5.** Variables affecting the odds of the presence of an upper age limit of 75 years or lower in phase 3 randomized controlled trials in gastric cancer (sensitivity analysis for the upper age limits of 85 years or lower)

| **Cancer stage**  Metastatic  Early/LAR  **Start date**  2009-2016  2017-2024  **Site in North America^3^**  No  Yes    **Site in Asia^3^**  No  Yes  **Site in Europe^3^**  No  Yes  **Chemotherapy**  No  Yes  **Targeted drug**  No  Yes  **Immunotherapy**  No  Yes  **Sample size**  ≤249  250-499  ≥500  **Primary sponsor**  Commercial  Non-commercial | **Adjusted odds ratio**  **(95% CI)**  Referent  1.35 (0.63-2.87)  Referent  1.60 (0.78-3.32)  Referent  0.09 (0.01-0.41)  Referent  2.16 (0.44-13.27)  Referent  0.45 (0.09-2.58)  Referent  0.89 (0.31-2.65)  Referent  1.03 (0.44-2.45)  Referent  1.48 (0.55-4.14)  Referent  1.09 (0.47-2.54)  1.38 (0.59-3.26)  Referent  2.10 (0.83-5.49) | **p**  -  0.43  -  0.20  -  **<0.001**  -  0.34  **-**  0.35  -  0.83  -  0.95  -  0.44  -  0.84  0.45  -  0.11 |
| --- | --- | --- |

**Additional Table 6.** Variables affecting the odds of excluding patients with ECOG score > 1 in phase 3 randomized controlled trials in gastric cancer (sensitivity analysis for variable ‘sample size’)

| **Cancer stage**  Metastatic  Early/LAR  **Start date**  2009-2016  2017-2024  **Site in North America^3^**  No  Yes    **Site in Asia^3^**  No  Yes  **Site in Europe^3^**  No  Yes  **Chemotherapy**  No  Yes  **Targeted drug**  No  Yes  **Immunotherapy**  No  Yes  **Sample size**  ≤249  250-499  ≥500  **Primary sponsor**  Commercial  Non-commercial | **Adjusted odds ratio**  **(95% CI)**  Referent  1.73 (0.79-3.90)  Referent  4.47 (2.11-9.85)  Referent  1.83 (0.53-6.64)  Referent  2.19 (0.60-7.92)  Referent  3.45 (0.97-12.99)  Referent  0.32 (0.08-1.07)  Referent  2.47 (0.95-6.93)  Referent  1.26 (0.44-3.82)  Referent  1.24 (0.58-2.62)  Referent  0.80 (0.31-2.07) | **p**  -  0.16  -  **<0.001**  -  0.33  -  0.22  **-**  0.05  -  0.06  -  0.06  -  0.67  -  0.57  -  0.64 |
| --- | --- | --- |

**Additional Table 7.** Variables affecting the odds of excluding patients with ECOG score > 1 in phase 3 randomized controlled trials in gastric cancer (sensitivity analysis replacing the trial’s start date with the registration date)

| **Cancer stage**  Metastatic  Early/LAR  **Start date**  2009-2016  2017-2024  **Site in North America^3^**  No  Yes    **Site in Asia^3^**  No  Yes  **Site in Europe^3^**  No  Yes  **Chemotherapy**  No  Yes  **Targeted drug**  No  Yes  **Immunotherapy**  No  Yes  **Sample size**  ≤249  250-499  ≥500  **Primary sponsor**  Commercial  Non-commercial | **Adjusted odds ratio**  **(95% CI)**  Referent  1.80 (0.81-4.17)  Referent  6.78 (3.15-15.35)  Referent  1.93 (0.55-7.18)  Referent  1.99 (0.53-7.37)  Referent  3.74 (1.01-14.58)  Referent  0.30 (0.07-0.99)  Referent  2.90 (1.08-8.52)  Referent  1.02 (0.35-3.16)  Referent  0.94 (0.38-2.31)  1.23 (0.49-3.08)  Referent  0.66 (0.24-1.81) | **p**  -  0.15  -  **<0.001**  -  0.30  -  0.30  **-**  **0.04**  -  **0.04**  -  **0.03**  -  0.96  -  0.89  0.65  -  0.41 |
| --- | --- | --- |

**Eligibility criteria concerning comorbidities in phase 3 randomized controlled trials in gastric cancer**

**Liver diseases**

In total – 170 (82.1%) trials

Criteria concerning aspartate/alanine aminotransferase:

Aspartate/alanine aminotransferase relative to upper limit of normal (ULN) – 105 (50.7%) trials; median cut-off - 2.5 ULN (range, 1.5-5 ULN)

Aspartate/alanine aminotransferase (IU/L) – 7 (3.4%) trials; median cut-off - 100 IU/L (range, 100-100 IU/L)

Aspartate/alanine aminotransferase relative to ULN in patients with liver metastases – 43 (20.8%) trials; median cut-off - 5 ULN (range, 5-5 ULN)

Criteria concerning bilirubin:

Bilirubin concentration relative to ULN – 99 (47.8%) trials; median cut-off – 1.5 ULN (range, 1-3 ULN)

Bilirubin concentration (mg/dL) – 15 (7.2%) trials; median cut-off – 1.5 mg/dL (range, 1.5-3 mg/dL)

Bilirubin concentration relative to ULN in patients with Gilbert syndrome – 7 (3.4%) trials; median cut-off – 3 ULN (range, 2-5 ULN)

Bilirubin concentration relative to ULN in patients with liver metastases – 5 (2.4%) trials; median cut-off – 2 ULN (range, 2-3 ULN)

Other criteria concerning liver diseases:

Hepatitis – 117 (56.5%) trials including: 1) HBV-DNA+, HCV-RNA+ and/or HBsAg+, HCVAg+ (n=88; 42.5%); 2) active disease defined as HBV-DNA+, HCV-RNA+ (n=20; 9.7%); 3) Other (n=9; 4.3%)

Vague eligibility criteria (e.g. adequate liver function) – 44 (21.3%) trials

Alkaline phosphatase (ALP) relative to ULN – 20 (9.7%) trials; median cut-off - 2.5 ULN (range, 1.5-6 ULN)

Cirrhosis – 15 (7.2%) trials

**Kidney diseases**

In total – 168 (81.2%) trials

Creatinine concentration relative to ULN – 68 (32.9%) trials; median cut-off – 1.5 ULN (range, 1-2 ULN)

Vague criteria (e.g.’adequate renal function’ – 69 (33.3%) trials

Creatinine clearance – 60 (29.0%) trials; median cut-off – 50 (range, 15-60)

Creatinine concentration (mg/dL) – 23 (11.1%) trials; median cut-off – 1.5 mg/dL (range, 1.2-2 mg/dL)

Glomerular filtration rate (GFR) – 8 (3.9%) trials; median cut-off – 50 (range, 30-60)

**Bone marrow function**

In total – 157 (75.8%) trials

Platelet count – 116 (56.0%) trials; median cut-off – 100,000/mm^3^ (range, 75,000-100,000/mm^3^)

Absolute neutrophil count – 107 (51.7%) trials; median cut-off – 1.5/mm^3^ (range, 1-3/mm^3^)

Hemoglobin – 96 (46.4%) trials; median cut-off value – 9 g/dL (range, 5-11 g/dL)

Vague eligibility criteria (e.g. ‘adequate bone marrow function’) – 40 (19.3%) trials

White blood cell count – 40 (19.3%) trials; median cut-off – 3,000/mm^3^ (range, 2-5/mm^3^)

**Prior/concurrent malignancies**

In total – 147 (71.0%) trials

Timeframe: 1) Concurrent malignancy (n=23; 11.1%); 2) Malignancy within the last 2 years (n=11; 5.3%); 3) Malignancy within the last 3 years (n=10; 4.8%); 4) Malignancy within the last 5 years (n=75; 36.2%); 5) Any history of malignancy (n=28; 13.5%)

Allowable exceptions: 1) Non-melanoma skin cancer (n=54; 26.1%); 2) Carcinoma in situ of the cervix uteri (n=51; 24.6%); 3) Carcinoma in situ of the bladder (n=18; 8.7%); 4) Breast carcinoma in situ (n=15; 7.2%); 5) Prostate cancer in situ (n=9; 4.3%); 6) Thyroid carcinoma in situ (n=6; 2.9%). Trials may have had multiple allowable exceptions.

**Cardiac diseases**

In total – 141 (68.1%) trials

Acute coronary syndrome – 87 (42.0%) trials

Heart failure – 85 (41.1%) trials

Exclusion of patients with NYHA class 2 - 22 (10.6%) trials

Exclusion of patients with NYHA class 3 - 33 (15.9%) trials

Exclusion of patients with heart failure with unspecified NYHA score – 37 (17.9%) trials

Arrhythmia – 72 (34.8%) trials

Vague eligibility criteria (e.g. ‘adequate cardiac function’) – 69 (33.3%) trials

Left ventricular ejection fraction (LVEF) – 29 (14.0%) trials; median cut-off – 50% (range, 40-55%)

**Psychiatric disorders**

In total – 75 (36.2%) trials

Unspecified psychiatric disorders – 49 (23.7%) trials

Severe/serious/uncontrolled disorders – 15 (7.2%) trials

Compliance/informed consent concerns – 11 (5.3%) trials

**Lung diseases**

In total – 74 (35.7%) trials

Interstitial lung disease (ILD)/non-infectious pneumoniae – 44 (21.3%) trials

Vague eligibility criteria (e.g. ‘adequate lung function’) – 22 (10.6%) trials

Lung fibrosis – 17 (8.2%) trials

Chronic obstructive pulmonary disease/emphysema – 9 (4.3%)

Other criteria – 7 (3.4%) trials

**Hypertension**

In total – 70 (33.8%) trials

Vague eligibility criteria (e.g. ‘uncontrolled blood pressure’) – 46 (22.2%) trials

Eligibility criteria with a specified cut-off for blood pressure:

Systolic blood pressure – 24 (11.6%) trials; median cut-off – 150 (range, 140-180)

Diastolic blood pressure – 24 (11.6%) trials; median cut-off – 100 (range, 90-100)

**Human immunodeficiency virus (HIV) infection**

In total – 57 (27.5%) trials

**Autoimmune diseases**

In total – 44 (21.3%) trials

**Stroke**

In total – 36 (17.4%) trials

**Thromboembolism**

In total – 23 (11.1%) trials

**Inflammatory bowel disease**

In total – 21 (10.1 %) trials

**Additional Table 8**. Trends in the use of the eligibility criteria concerning selected comorbidities over time (sensitivity analysis by the trial’s registration date).

| Eligibility criterion | Trials registered between 2009 and 2016, % | Trials registered between 2017 and 2024, % | p |
| --- | --- | --- | --- |
| Liver disorders  Renal diseases^1^  Renal diseases^2^  Renal diseases^3^ | 45.3%  46.4%  53.7%  54.1% | 54.7%  53.6%  46.3%  45.9% | 0.28  0.40  0.40  0.24 |

^1^All eligibility criteria concerning renal diseases. ^2^Eligibility criteria with the cut-off for creatinine clearance 60 or creatinine level ≤1.0 ULN. ^3^Eligibility criteria with the cut-off for creatinine clearance 45-60 or creatinine level ≤1.0-1.25 ULN.

**Additional Table 9**. Trends in the use of the eligibility criteria concerning brain metastases over time (sensitivity analysis by the trial’s registration date).

| Eligibility criterion | Trials registered between  2009 and 2016, % | Trials registered between 2017 and 2024, % | p |
| --- | --- | --- | --- |
| BM, overall^1^  Strict exclusion^2^  Conditional exclusion^3^ | 50.5%  71.1%  35.8% | 49.5%  28.9%  64.2% | 0.48  **0.001**  **0.04** |

^1^All eligibility criteria related to brain metastases. ^2^Criteria excluding patients with any form of brain metastases; ^3^Criteria excluding patients with active/symptomatic/untreated brain metastases. Abbreviations: BM, brain metastases.
